# A Novel Disulfidptosis‐Related Diagnostic Gene Signature and Differential Expression Validation in Ischaemic Cardiomyopathy

**DOI:** 10.1111/jcmm.70475

**Published:** 2025-03-11

**Authors:** Xin Tan, Shuai Xu, Yiyao Zeng, Fengyi Yu, Zhen Qin, Ge Zhang, Jili Fan, Xiaohong Bo, Junnan Tang, Huimin Fan, Yafeng Zhou

**Affiliations:** ^1^ Department of Cardiology The Fourth Affiliated Hospital of Soochow University, Suzhou Dushu Lake Hospital, Medical Center of Soochow University Suzhou China; ^2^ Institute for Hypertension Soochow University Suzhou China; ^3^ Department of Cardiology The First Affiliated Hospital of Zhengzhou University Zhengzhou China; ^4^ Henan Province Key Laboratory of Cardiac Injury and Repair Zhengzhou China; ^5^ Henan Province Clinical Research Center for Cardiovascular Diseases Zhengzhou China; ^6^ Department of Cardiovascular Disease Taihe County People's Hospital Fuyang China; ^7^ Center of Translational Medicine and Clinical Laboratory, the Fourth Affiliated Hospital to Soochow University, Suzhou Dushu Lake Hospital Suzhou China

**Keywords:** diagnostic model, disulfidptosis‐related genes, immune cell infiltration, ischaemic cardiomyopathy

## Abstract

Ischaemic cardiomyopathy (IC) predominantly arises from prolonged deprivation of oxygen in the coronary arteries, resulting in compromised cardiac contractility or relaxation. This study investigates the role of disulfidptosis‐associated genes (DiGs) in IC. Through the analysis of datasets GSE5406 and GSE57338, we explored the association between DiGs and immune characteristics to identify crucial genes contributing to IC development. The support vector machine model emerged as the most effective, identifying key genes such as MYH9, NUBPL, MYL6, MYH10 and NCKAP1. Validation with independent datasets GSE57345, GSE48166 and single‐cell GSE145154 further supported these findings, demonstrating high predictive accuracy. Experimental validation in an IC mouse model, using Western blot, immunohistochemistry and RT‐qPCR, confirmed the altered expression of these core genes in myocardial ischaemic regions. This research not only elucidates the significance of DiGs in IC but also underscores the diagnostic potential of identified core genes.

**Abbreviations**
AMI: acute myocardial infarction
AUC: Area Under the Curve
ceRNA: Competing Endogenous RNA
CNV: Copy number variation
DCA: Decision Curve Analysis
DiGs: disulfidptosis‐related genes
FDR: False discovery rate
GEO: Gene Expression Omnibus
GLM: Generalised Linear Model
GSVA: Gene Set Variation Analysis
GYS1: Glycogen synthase 1
HIF‐1α: hypoxia‐inducible factor‐1α
HK2: hexokinase 2
IC: Ischaemic cardiomyopathy
LDH: lactate dehydrogenase
LVEF: left ventricular ejection fraction
LVFS: left ventricular fractional shortening
MYH10: Myosin‐10
MYH9: Myosin‐9
MYL6: Myosin light polypeptide 6
NCKAP1: NCK associated protein 1
NUBPL: Iron–Sulphur Protein NUBPL
PYGM: Glycogen phosphorylase, muscle form
RF: Random Forest
ROC: Receiver Operating Characteristic
SVM: Support Vector Machine
XGB: Extreme Gradient Boosting

## 1 Introduction

Ischaemic cardiomyopathy (IC) is a cardiovascular disease caused by prolonged myocardial ischaemia [1]. With the advancement of interventional treatments, the survival rate of patients with acute myocardial infarction has significantly improved, but the incidence of IC is increasing yearly [2, 3, 4]. The main clinical manifestations of IC include angina, heart failure and arrhythmias. Current treatment strategies focus on improving myocardial ischaemia, reducing myocardial injury and preventing heart failure [5, 6]. Additionally, with the in‐depth study of the pathological mechanisms of IC, new diagnostic and therapeutic targets, such as cell death‐related genes, provide new directions for the diagnosis and treatment of IC in the future.

The pathogenesis of IC is closely associated with various cell death modes in myocardial cells [7, 8, 9, 10]. Recent research has discovered that disulfidptosis is a new form of cell death, attracting growing attention [11, 12]. The core mechanism of disulfidptosis involves the excessive accumulation of disulfide bonds, leading to the collapse of the cytoskeleton and subsequent cell death. This process mainly relies on a protein called SLC7A11, which is involved in the synthesis and transport of glutathione, thereby affecting the cell's redox balance [12, 13]. This novel cell death mechanism has attracted widespread attention in cancer research [14]. However, the impact of disulfidptosis extends beyond cancer. Studies have indicated that disulfidptosis‐related genes can serve as diagnostic markers for ulcerative colitis and chronic obstructive pulmonary disease [15, 16]. Interestingly, recent findings suggest a potential link between disulfidptosis and glycogen metabolism. Disulfidptosis‐related genes (DiGs), such as GYS1, play a pivotal role in glycogen accumulation [17], a hallmark of myocardial hibernation often observed in ischaemic cardiomyopathy [18, 19]. During ischaemic conditions, myocardial cells experience prolonged disruptions in cytoskeletal structure and metabolic homeostasis, which may share mechanistic parallels with disulfidptosis [20]. Understanding the involvement of DiGs in IC may provide novel insights into the pathogenesis of this disease and reveal new therapeutic targets.

Bioinformatics is rapidly evolving, playing a crucial role in identifying essential diagnostic genes for various diseases [21]. Machine learning has been widely adopted in the medical field, especially in areas such as disease diagnosis, prediction and treatment. This approach allows computers to learn from data and improve their performance. The process includes data collection and preprocessing, feature extraction and algorithm selection, model training using the training data, evaluating and optimising model performance and finally, deploying the model with ongoing monitoring and maintenance to ensure its effectiveness. Predicting disease biomarkers is a vital application of machine learning, aiding physicians in making more accurate diagnostic and treatment decisions, ultimately improving cure rates and patient outcomes [10].

In this study, we analysed the role of DiGs in the development of IC using bioinformatics methods. First, using the Gene Expression Omnibus (GEO) database, we identified DiGs between IC patients and healthy individuals. Next, based on the expression profiles of DiGs, we classified IC into two clusters related to DiGs, conducted immune infiltration analysis, identified core genes through machine learning, validated them using external datasets and determined their distribution characteristics in single‐cell datasets. Finally, we validated our findings through in vivo experiments. In summary, by deeply exploring the specific mechanisms of disulfidptosis in IC, we can provide new ideas and methods for early diagnosis, prognosis evaluation and therapeutic intervention of this disease.

## 2 Materials and Methods

### 2.1 Data Source

**FIGURE 1:**
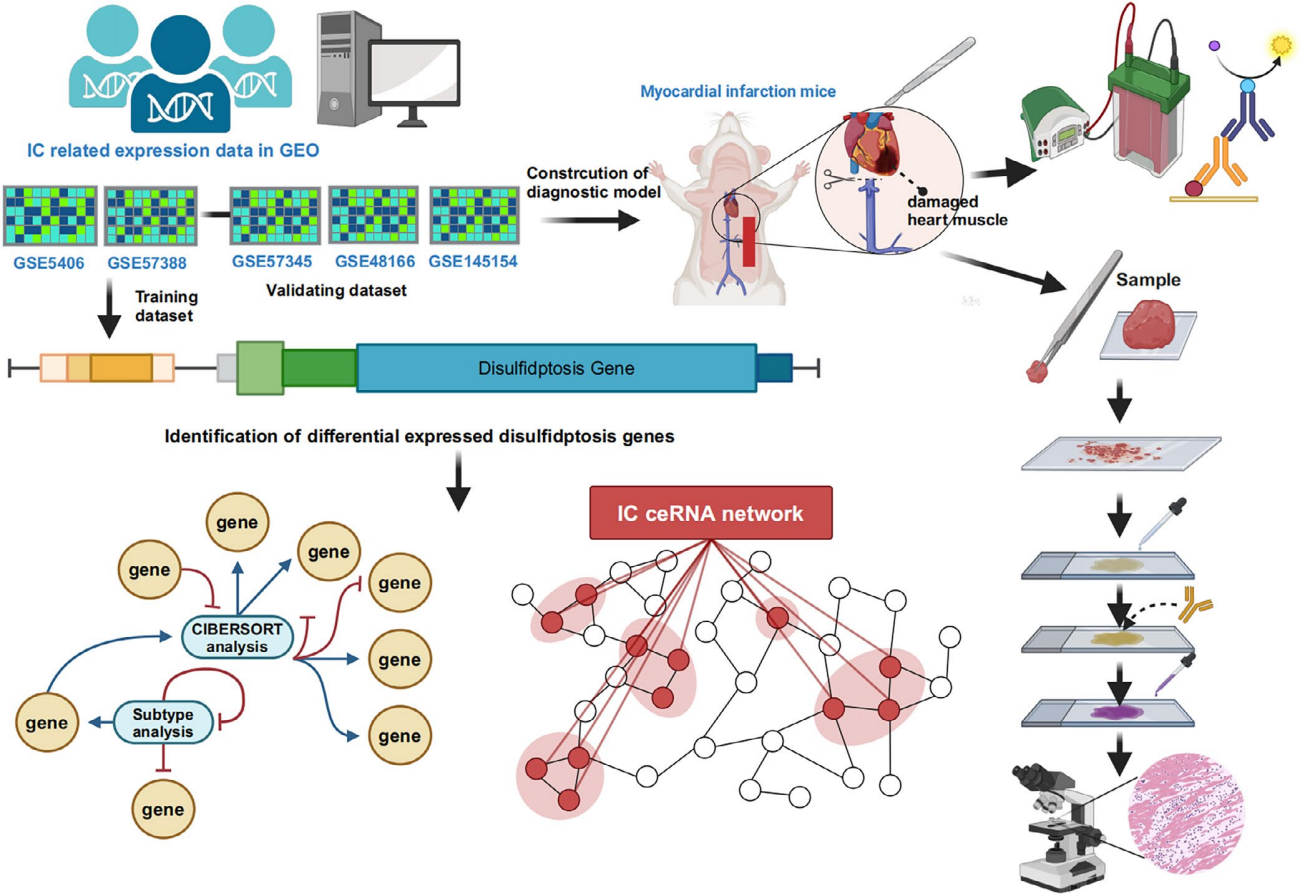
The study design.

Datasets GSE57345 [22], GSE57338 [23], GSE48166, GSE5406 [24], GSE29532 [25] and GSE123342 [26] were retrieved from the GEO database, as listed in Table S1 [27]. Employing the sva package in R version 4.3.2, data from GSE57338 and GSE5406 underwent batch correction and were combined (152 control and 203 IC samples) [28]. The datasets GSE57345 and GSE48166 served as validation sets (Figure 1).

### 2.2 Selection of Differentially Expressed DiGs

Twenty‐three DiGs were selected based on a previous study for further analysis [11, 12]. The t‐test was used to evaluate the expression differences of DiGs between the IC group and the control group, identifying statistically significant genes (*p* < 0.05). Display differentially expressed genes in a heatmap [29, 30].

### 2.3 Cluster Analysis Based on Differentially Expressed DiGs

Cluster analysis was performed with ConsensusClusterPlus, focusing on the DiGs within the training set [31]. Following this, the training dataset underwent evaluation through gene set variation analysis (GSVA) [32].

### 2.4 Machine‐Learning Methods

For the predictive identification of IC, we employed various machine‐learning methods to develop comprehensive models. These models comprised an extreme gradient boosting (XGB) [21], random forest (RF) [33], generalised linear model (GLM) [34] and support vector machine (SVM) [35]. We used the DALEX package to visualise the distribution of residuals in machine‐learning models. The most effective machine‐learning model was chosen based on performance, leading to the identification of the top five critical diagnostic genes. The performance metric, area under the curve (AUC) of the receiver operating characteristic (ROC) curves, was illustrated with the ‘pROC’ package in R.

### 2.5 Nomogram Construction and Validation

To enhance validation and practical application of the predictive capability, construct a nomogram using the ‘RMS’ package. In this model, each predictor was assigned a distinct score, with the ‘total score’ representing the sum of these individual scores. Calibration curves and decision curve analysis (DCA) were employed to evaluate the predictive accuracy of the nomograms.

### 2.6 Correlation of Core Genes With IC Age and Gender and Longitudinal Analysis

The GSE57338 dataset contains gene expression profiles of myocardial tissue samples along with complete age and gender information. Pearson correlation analysis was used to assess the linear relationship between core gene expression levels and sample age. The Wilcoxon rank‐sum test was used to analyse the expression differences of core genes between different gender groups. The ggplot2 package in R was used to create scatter plots, regression fitting curves and histograms to visually display the relationships between gene expression and age or gender. Longitudinal expression patterns of DiGs were analysed using GSE29532 (acute STEMI: six time points, 0, 2, 12, 24, 36 and 48 h) and GSE123342 (long‐term MI follow‐up: Day 0, Day 30 and Year 1).

### 2.7 Analysis of Immune Cell Infiltration in IC

The analysis of 22 immune cell types was performed using the CIBERSORT algorithm (https://cibersort.stanford.edu/). This process involved Monte Carlo sampling for ordering, which generated *p*‐values for the deconvolution of each sample. Significance was determined at *p* < 0.05, ensuring that the sum of the proportions of the 22 immune cell types in each sample equalled 1 [36].

### 2.8 ceRNA Network Construction

The construction of a ceRNA network involved predicting miRNA–miRNA interactions using TargetScan, miRDB and miRanda databases. Prediction of miRNA–lncRNA interactions was obtained from the SpongeScan database.

### 2.9 Animal

We purchased 8‐week‐old male and female C57 mice were purchased from the Animal Center of Zhengzhou University Medical College, Henan Province (SCXK 2022–0001). All procedures were meticulously conducted in compliance with the ‘Guidelines of the Chinese Animal Research Council’ and ‘Animal Care Guidelines’ to minimise the suffering of animals. Mice were accommodated in well‐ventilated rooms, maintained at a moderate temperature (22C ± 2°C) and suitable humidity (55% ± 5%) and subjected to a 12‐h light/dark cycle. They had unrestricted access to food and water. Following a week of acclimatisation on standard laboratory mouse feed, the mice were randomly allocated into two groups: a control group (*n* = 16, 8 males and 8 females) and an IC group (*n* = 16, 8 males and 8 females). The IC mouse model was established using a method previously described by us [37, 38], the duration of the left anterior descending (LAD) artery ligation procedure was strictly controlled within 10–12 min for all animals, and to ensure consistency, all surgical procedures were performed by the same trained operator. For anaesthesia, mice were anaesthetised using 2% isoflurane administered via inhalation. After the mouse is anaesthetised and intubated, a thoracotomy is performed at the third or fourth intercostal space on the left side of the chest to expose the heart. The LAD artery is identified between the left atrial appendage and the pulmonary cone, using the main trunk of the left coronary artery as a marker. The LAD artery is then ligated with a 6–0 suture needle. Successful ligation is confirmed when tissues below the ligation area turn white. Finally, the chest is closed layer by layer. Mice in the sham operation group undergo the same thoracotomy procedure without LAD ligation. After 1 week of postoperative observation, all mice were euthanised as follows: Prior to cervical dislocation, each animal was anaesthetised with 2% isoflurane inhalation to minimise distress, and cervical dislocation was subsequently performed. The body weight of the mice was measured at the beginning of the experiment (presurgery) and before euthanasia. The body weight of the mice was measured using an electronic balance, recorded to one decimal place, to assess physiological changes during the experimental process.

### 2.10 Chemicals and Materials

Sodium pentobarbital (Sigma, 57–33‐0, USA), and antibodies: MYH9 (Rabbit, 11,128‐1‐AP, Proteintech, China), NUBPL (Rabbit, 17,393‐1‐AP, Proteintech, China), MYL6 (Mouse, 68,142‐1‐Ig, Proteintech, China), NCKAP1 (Rabbit, 12,140‐1‐AP, Proteintech, China), MYH10 (Rabbit, 19,673‐1‐AP, Proteintech, China), β‐actin (Mouse, 60,008‐1‐Ig, Proteintech, China), goat anti‐rat IgG (HRP Conjugate) (98,164, CST, USA) and goat anti‐mouse IgG (HRP Conjugate) (91,196, CST, USA).

### 2.11 Echocardiography for Cardiac Function Assessment

Cardiac function in mice was assessed using a small animal ultrasound imaging system (FUJIFILM VisualSonics, Canada). Before the experiment, 2% isoflurane anaesthesia was administered to minimise mouse activity and maintain stable vital signs. Two‐dimensional images of the left ventricle were obtained in the short‐axis or long‐axis views, and the systolic and diastolic motions were recorded using M‐mode. Indicators such as heart rate, left ventricular ejection fraction (LVEF), fractional shortening (LVFS), left ventricular end‐diastolic diameter (LVEDD) and left ventricular end‐systolic diameter (LVESD) were measured using the ultrasound system to evaluate cardiac function. Statistical analysis was conducted using the average values of three cardiac cycles. Simultaneously, the heart rate was monitored using ultrasound equipment and recorded as the number of beats per minute. LVEF% = (LVEDD^3^—LVESD^3^)/LVEDD^3^. LVFS% = (LVEDD—LVESD)/(LVEDD).

### 2.12 Lactate Dehydrogenase (LDH) ELISA Assay

One week after surgery, mice were euthanised, 1 mL of peripheral blood was collected, centrifuged at 3000 rpm for 10 min at 4°C and the serum supernatant was separated. Standards and samples were added to a 96‐well plate according to the instructions of the LDH assay kit (12,240, MEIMIAN, China). Enzyme conjugates were added, incubated at room temperature for 30 min and washed to remove unbound substances. A chromogenic substrate was added, incubated in the dark for 15 min and the reaction was stopped. Absorbance values were measured at 450 nm using a microplate reader. The LDH concentrations were determined from the standard curve, and the differences between the IC group and the control group were analysed.

### 2.13 Histopathological Examination of Myocardium

Mouse cardiac tissue was fixed in 4% paraformaldehyde for 24 h, then subjected to gradient dehydration, paraffin embedding and sectioning at a thickness of 4 μm. The sections were deparaffinised with xylene, rehydrated through a graded ethanol series, stained with haematoxylin for 5 min, differentiated with 1% hydrochloric acid alcohol for 1 s and blued with ammonia water. Subsequently, sections were stained with eosin for 1–2 min, quickly dehydrated, cleared and cover slipped. The arrangement of myocardial cells and abnormalities in tissue structure were observed under a light microscope.

The fixed heart tissue was processed through gradient dehydration, paraffin embedding, sectioning and Masson's trichrome staining to evaluate myocardial fibrosis. The sections were stained with haematoxylin for nuclei, acidic fuchsin for cytoplasm, differentiated using a phosphomolybdic acid solution and stained with aniline blue for collagen fibres. After dehydration and mounting, observations were made under a microscope. The infarct area was quantified by measuring the percentage of the infarcted area relative to the total left ventricular area in each section.

Immunohistochemical staining (IHC) was performed using primary antibodies MYH9 (1:500), NUBPL (1:500), MYL6 (1:1000), MYH10 (1:200) and NCKAP1 (1:500). After deparaffinisation and hydration, sections were treated with 3% hydrogen peroxide to block endogenous peroxidase activity for 10 min. Sections were blocked with 1% bovine serum albumin, followed by the addition of primary antibodies and incubation overnight at 4°C. The next day, the sections were washed three times with PBS, followed by the addition of HRP‐conjugated secondary antibodies and incubation at room temperature for 30 min. DAB was used for chromogenic detection (with the time adjusted according to staining intensity), followed by haematoxylin counterstaining, gradient dehydration, clearing, mounting and observation under a microscope.

### 2.14 Detecting the Expression Levels of Key Disulfidptosis Proteins in Myocardial Tissue

Proteins were extracted, quantified and denatured from myocardial tissue homogenates, followed by electrophoresis and subsequent membrane transfer. Block with 5% skim milk for 2 h. Subsequently, antibodies against MYH9 (1:1000), NUBPL (1:1000), MYL6 (1:1000), MYH10 (1:1000) and NCKAP1 (1:2000) were applied sequentially and incubated on a shaker at 4°C overnight. Wash the membrane (15 min each time, three times in total), incubate with the secondary antibody at room temperature for 1 h and then wash the membrane again as described above before exposure (tanon5200, China).

### 2.15 Reverse Transcription‐Quantitative Polymerase Chain Reaction (RT‐qPCR)

Total RNA was extracted from the myocardial tissue of each group of mice using Trizol reagent following the manufacturer's instructions, and the purity and concentration were measured using an N50 UV spectrophotometer (Implen, Germany). Each sample used 1 μg of RNA, which was reverse‐transcribed into cDNA using a reverse transcription kit (TransGen Biotech, Beijing, China) following the provided protocol. Subsequently, real‐time quantitative PCR was performed using a SYBR Green qPCR kit (Selleck, Houston, TX, USA) in a 20 μL reaction system, including 10 μL of 2× SYBR Green mix, 1 μL each of forward and reverse primers, 2 μL of cDNA template and 6 μL of deionised water. All primers were synthesised by Suzhou Jinweizhi Company. The qPCR conditions were as follows: 95°C for 30 s for initial denaturation, followed by 40 cycles of 95°C for 5 s (denaturation) and 60°C for 30 s (annealing). β‐Actin was used as an internal control, and relative expression levels were calculated using the 2^−ΔΔCt^ method to measure mRNA expression levels of HIF‐1α, HK2, GYS1, PYGM, MYH9, NUBPL, MYL6, MYH10, NCKAP1 and β‐actin (Table 1).

**TABLE 1:** Primer pairs used in this study.

Primer name	Primer sequence (5′‐3′)
MYH9‐F	GGCCCTGCTAGATGAGGAGT
MYH9‐R	CTTGGGCTTCTGGAACTTGG
NUBPL‐F	GTTGGCTTGTTAGATGTGGATGT
NUBPL‐R	GCGCAGTCTCTTCAACCAAAA
MYL6‐F	CCAGTGTGGGGATGTGATGC
MYL6‐R	ATGACGGATTTCAGCACCCAT
MYH10‐F	GGAATCCTTTGGAAATGCGAAGA
MYH10‐R	GCCCCAACAATATAGCCAGTTAC
NCKAP1‐F	AGTCGGACACTATGCCTTGTG
NCKAP1‐R	GCGGTAGCCTCCGTATTTAGC
HIF‐1α‐F	ACCTTCATCGGAAACTCCAAAG
HIF‐1α‐R	CTGTTAGGCTGGGAAAAGTTAGG
PYGM‐F	CTTAGCCGGAGTGGAAAATGT
PYGM‐R	GTAATCTCTCGGAGTAGCCACA
GYS1‐F	GAACGCAGTGCTTTTCGAGG
GYS1‐R	CCAGATAGTAGTTGTCACCCCAT
HK2‐F	TGATCGCCTGCTTATTCACGG
HK2‐R	AACCGCCTAGAAATCTCCAGA
β‐Actin‐F	GGCTGTATTCCCCTCCATCG
β‐Actin‐R	CCAGTTGGTAACAATGCCATGT

### 2.16 Single‐Cell RNA‐Seq (scRNA‐Seq) Analysis

We analysed single‐cell RNA‐sequencing (GSE145154) data using the R package ‘Seurat’ [39]. To ensure the inclusion of high‐quality cell data, inclusion criteria involved genes expressed in at least three single cells, while exclusion criteria included genes with counts lower than 200 or exceeding 10,000 reads per cell, and cells with mitochondrial gene ratios exceeding 20%. The ‘harmony’ R package was employed to remove batch effects between samples [40]. The scRNA‐Seq data were normalised using the ‘Seurat’ R package. Following normalisation, the data were converted into a Seurat object and the ‘FindVariableFeatures’ function was utilised to identify the top 2000 highly variable genes. Subsequently, principal component analysis (PCA) was performed on the expression matrix of variable genes. Clustering was conducted using the ‘FindClusters’ function, and dimensionality reduction and visualisation were carried out using the ‘RunTSNE’ function. The ‘FindAllMarkers’ function was used to identify cluster‐specific markers and the ‘singleR’ package was employed to automatically reannotate all cell clusters, with annotations corrected using the CellMarke2.0r database [41].

### 2.17 Statistical Methods

The data were analysed statistically using GraphPad Prism 9.0, and all data are shown as mean ± standard deviation. An independent samples t‐test was used. *p < 0.05, **p < 0.01 and ns (nonsignificant).

## 3 Results

### 3.1 DiGs Expression and Immune Infiltration Analysis in IC Patients

**FIGURE 2:**
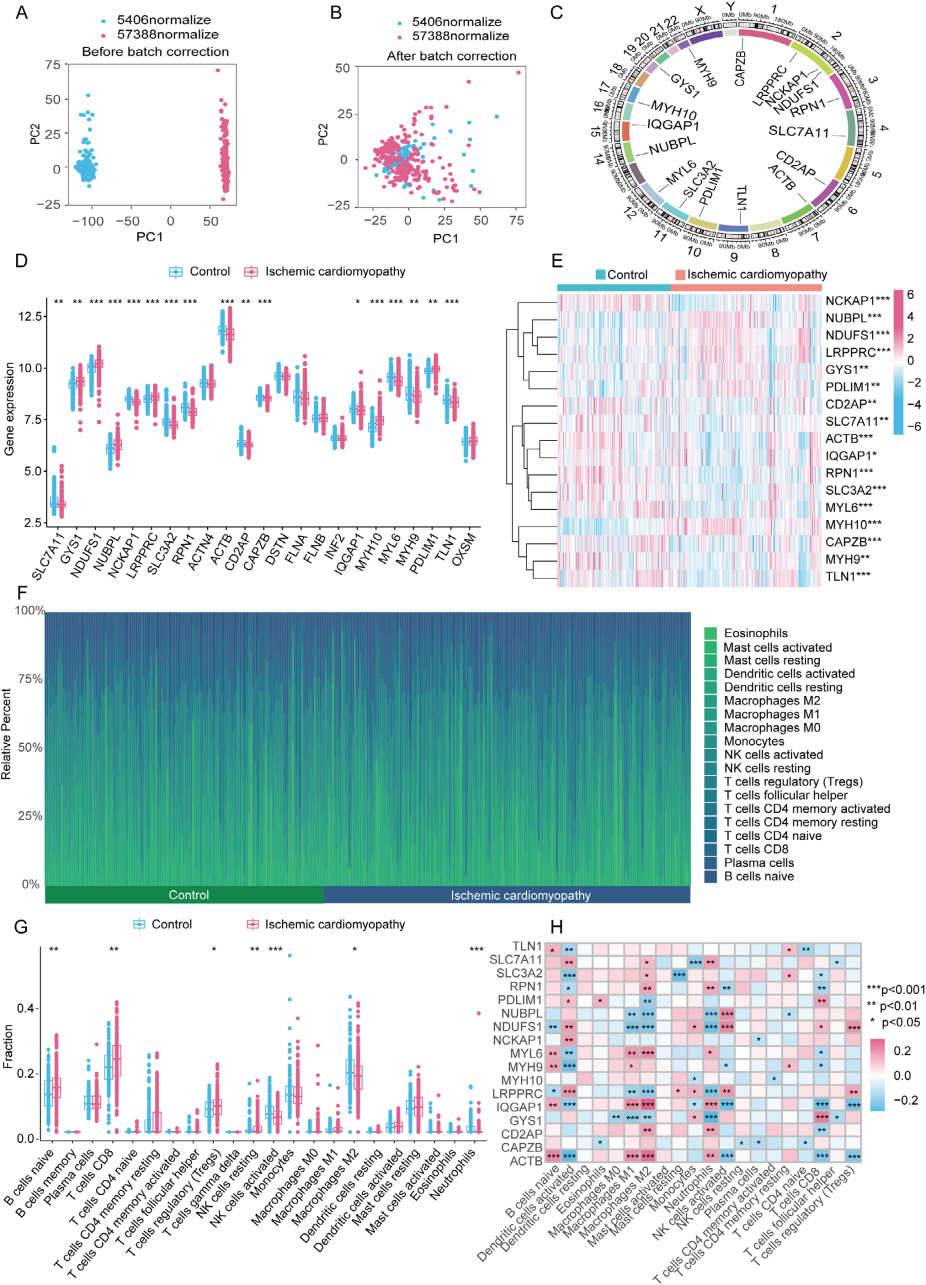
DiGs and immune infiltration analysis in IC. (A and B) Comparison of the dataset before and after removing batch effects. (C) The spatial distribution of CNV in DiGs across 23 chromosomes. (D and E) Differential expressions of 17 DiGs. (F) The relative abundance of immune cells between IC and Control. (G) Difference in immune infiltration. (H) The correlation analysis between 17 DiGs and immune cells. (*p ≤ 0.05, **p ≤ 0.01, ***p ≤ 0.001.)

The training set was obtained by merging and processing the GSE5406 and GSE57338 datasets (Figure 2A,B). The locations of the Copy number variation (CNV) changes for 17 DiGs on 23 chromosomes are shown in Figure 2C. We examined the expression profiles of 17 DiGs within IC and control samples, utilising the merged dataset. Comparative analysis revealed heightened expression levels on GYS1, NDUFS1, NUBPL, LRPPRC, MYH10 and PDLIM1, alongside diminished expression on SLC7A11, NCKAP1, SLC3A2, RNP1, ACTB, CD2AP, CAPZB, IQGAP1, MYL6, MYH9 and TLN1 (Figure 2D,E).

Following the initial setup, immune infiltration analysis was performed on both control and IC patient groups. Immune infiltration results indicate significant differences in naive B cells, resting NK cells, activated NK cells and M2 macrophages (Figure 2F,G). Additionally, correlation analysis highlighted disulfidptosis in regulatory M1 macrophages, naive B cells, CD8+ T cells, activated NK cells and M2 macrophages (Figure 2G).

### 3.2 Identification of IC Disulfidptosis Cluster

**FIGURE 3:**
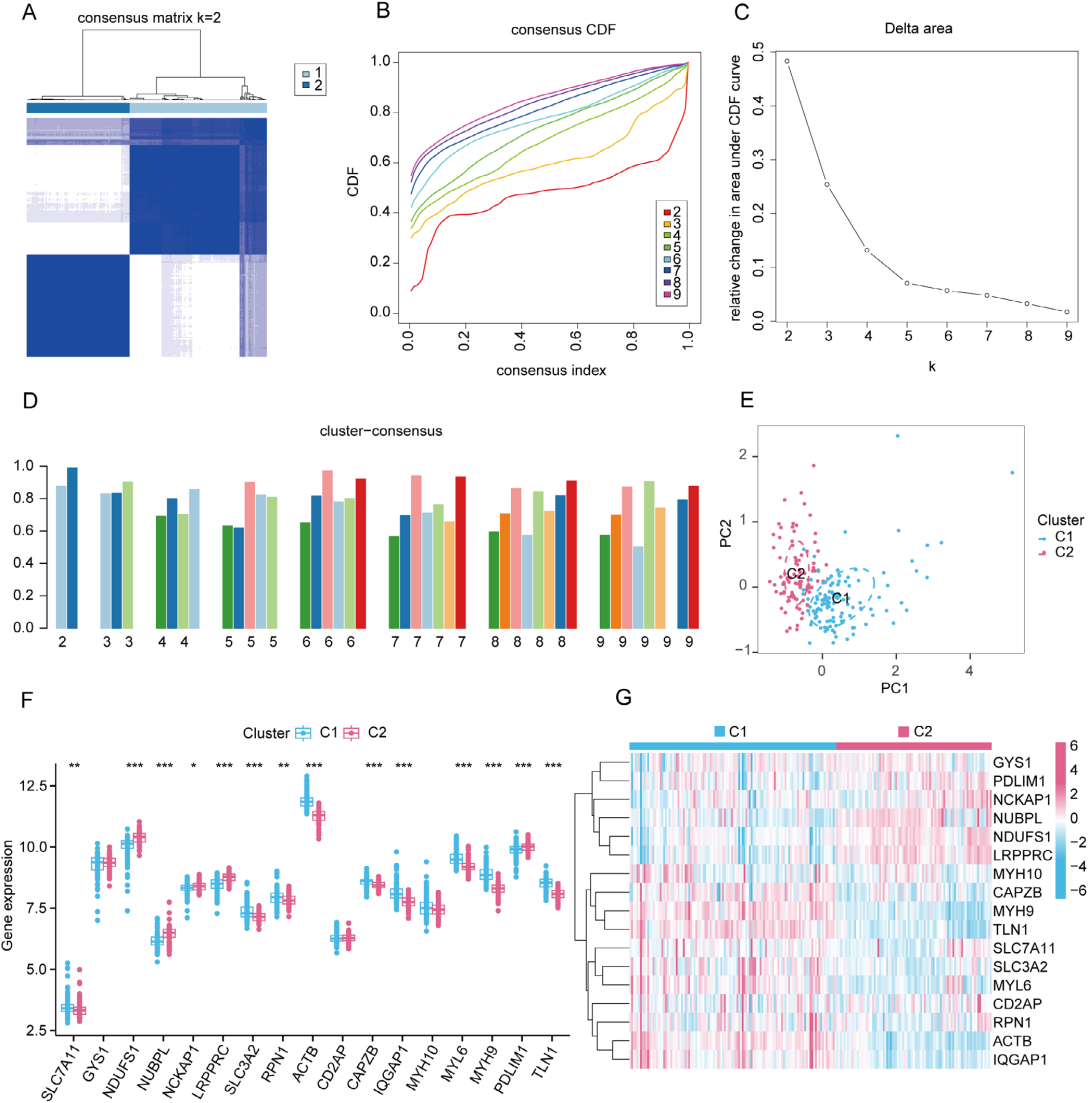
Identification of DiG molecular clusters in IC. (A) Consensus clustering matrix when *k* = 2. (B‐D) Representative cumulative distribution function (CDF) curves (B), CDF delta area curves (C) and the score of consensus clustering (D). (E) T‐SNE visualises the distribution of two clusters. (F) Boxplots showed the expression of 17 DiGs between two disulfidptosis clusters. (G) The expression patterns of 17 DiGs were presented in the heatmap. (*p ≤ 0.05, **p ≤ 0.01, ***p ≤ 0.001.)

The consensus index showed stable fluctuations within the range of 0.2–0.6 (Figures 3A,B and S1). As the number of clusters (k) varied from 2 to 9, the area under the CDF curve indicated significant differences between clusters (k and k‐1) (Figure 3C). Notably, a high consistency score (> 0.9) was achieved exclusively at *k* = 2, as detailed in Figure 3D. Following this, the consensus matrix heatmap facilitated the division of 203 patients into two distinct clusters: Cluster 1 (*n* = 116) and Cluster 2 (*n* = 87) (Figure 3E). Visualisation through t‐distributed random neighbourhood embedding (t‐SNE) highlighted pronounced disparities between the two clusters (Figure 3F). Importantly, Cluster 1 showed elevated expression of SLC7A11, SLC3A2, RPN1, ACTB, CAPZB, IQGAP1, MYL6, MYH9 and TLN1. Conversely, Cluster 2 exhibited increased expression of NDUFS1, NUBPL, NCKAP1, LRPPRC and PDLIM1 (Figure 3G).

Compared to Cluster 1, Cluster 2 has a higher proportion of M2 macrophages, CD8+ T cells, activated dendritic cells and naive B cells (Figures S2 and S3). Using GSVA, we identified cluster‐specific DEGs that underscored functional disparities between the clusters. Notably, Cluster 2 showed upregulation in pathways like natural killer cell‐mediated cytotoxicity and ERBB signalling pathway. Conversely, Cluster 1 was characterised by increased activity in oxidative phosphorylation, butanoate metabolism and aminoacyl‐tRNA biosynthesis (Figure S4).

Additionally, functional enrichment analysis revealed distinct biological processes linked with each cluster. Cluster 1 showed enrichment in biological processes such as positive regulation of macrophage differentiation and cytokine production involved in immune responses. In contrast, Cluster 2 demonstrated enrichment in processes including protein localisation to the phagophore assembly site and ATP transport (Figure S5).

### 3.3 Machine Learning Model Construction and Evaluation

**FIGURE 4:**
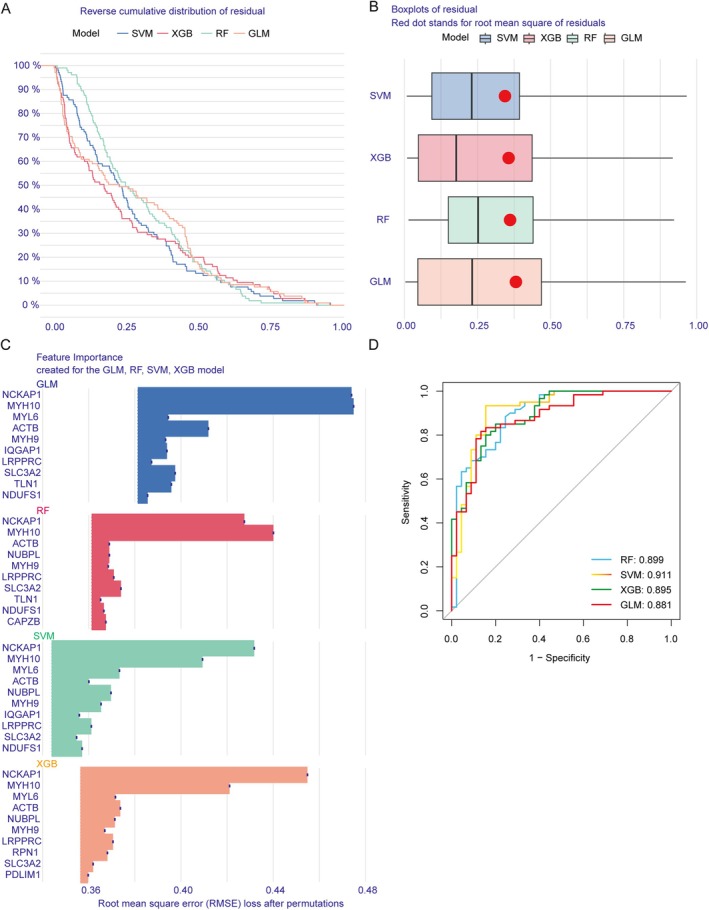
Construction and evaluation of SVM, RF, GLM and XGB machine models. (A) Cumulative residual distribution of each machine‐learning model. (B) The residuals of each machine‐learning model. (C) The important features in SVM, RF, GLM and XGB machine models. (D) ROC analysis of four machine‐learning models based on fivefold cross‐validation in the testing cohort.

Model residuals for each were visualised in the training dataset using the ‘DALEX’ software package, with the SVM model showing relatively minor residual differences (Figure 4A,B). The top 10 significant variables for each model were determined based on the root mean squared error (RMSE) (Figure 4C). The SVM model exhibited the highest AUC, with values as follows: SVM (AUC = 0.911), RF (AUC = 0.899), XGB (AUC = 0.895) and GLM (AUC = 0.881) (Figure 4D). This result underscores the SVM model's superior ability to differentiate between patient clusters. From the SVM model, the top five variables identified as key predictors for further analysis were MYH9, NUBPL, MYL6, MYH10 and NCKAP1.

**FIGURE 5:**
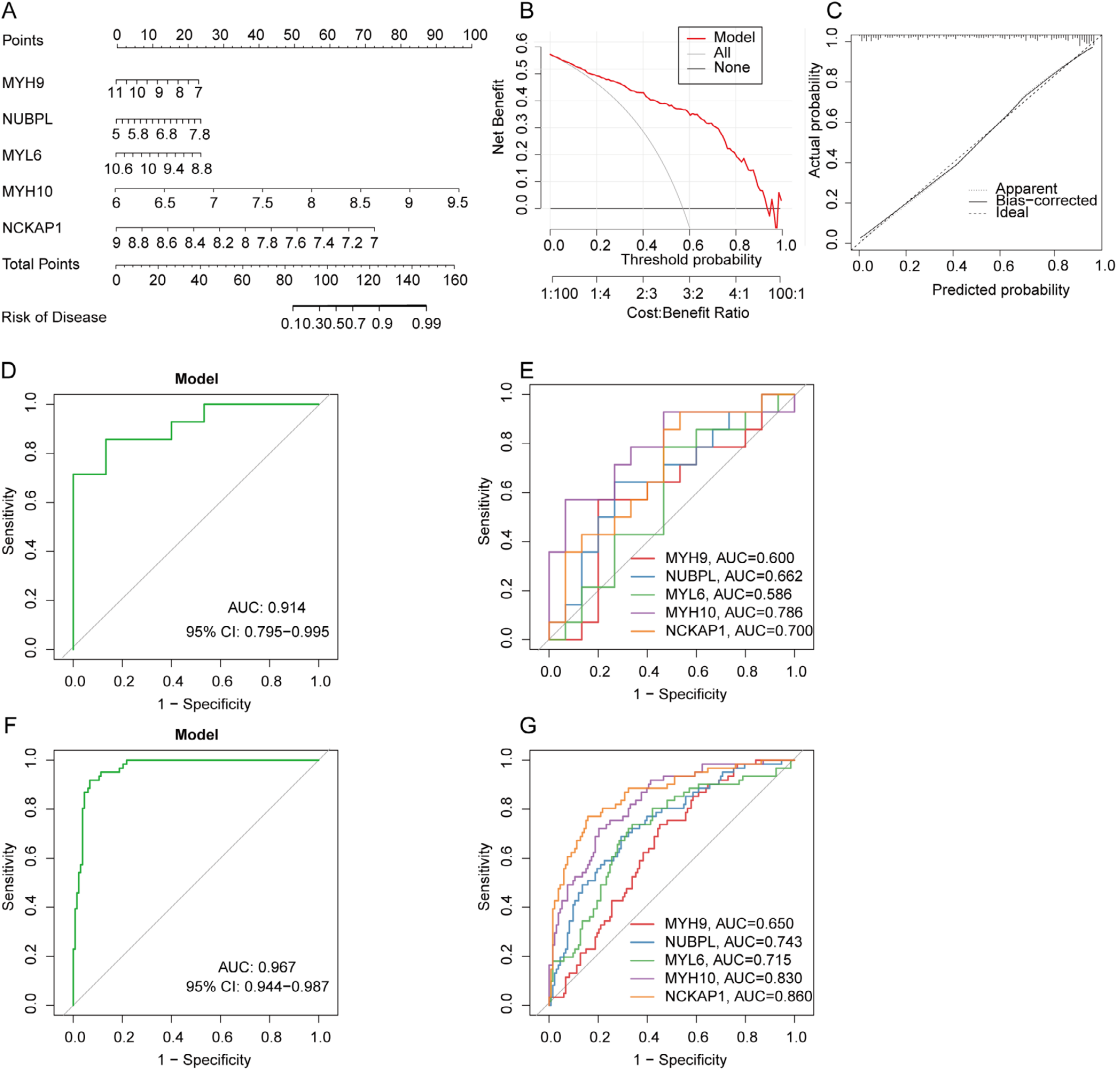
Validation of the five‐gene–based SVW model. (A) Construction of a nomogram for predicting the risk of IC clusters based on the five‐gene–based SVW model. (B, C) Construction of calibration curve (B) and DCA (C) for assessing the predictive efficiency of the nomogram model. (D, E) ROC analysis of the five‐gene–based SVW model based on fivefold cross‐validation in GSE48166. (F, G) ROC analysis of the five‐gene–based SVW model based on fivefold cross‐validation in GSE57345.

We developed a nomogram to evaluate the predictive efficiency of the SVM model across 203 IC cases (Figure 5A). The calibration curve showed minimal discrepancy between actual and predicted risks within the IC cluster (Figure 5B). DCA highlighted the nomogram's high accuracy, offering valuable insights for clinical decision‐making (Figure 5C). Additionally, the predictive capability of core biomarkers was validated using datasets GSE48166 and GSE57345. ROC curves revealed that the predictive model, incorporating the five core markers, performed effectively, with AUCs of 0.914 (GSE48166) and 0.967 (GSE57345), demonstrating the model's efficacy in distinguishing IC patients from healthy individuals (Figure 5D–G).

### 3.4 Correlation Between Core Genes and Clinical Features

The expression levels of MYH9 and MYL6 were significantly negatively correlated with age, with correlation coefficients of −0.27 (*p* = 0.0092) and −0.21 (*p* = 0.039), respectively, indicating a decrease in their expression levels with increasing age. MYH10, NUBPL and NCKAP1 expression levels were not significantly correlated with age (*p* > 0.05). The expression levels of core genes showed no significant differences between genders (*p* > 0.05) (Figure S6). GSE29532 revealed early upregulation of NUBPL and MYL6 at 2 h and peak expression of MYH9 and MYH10 at 24 h. GSE123342 demonstrated elevated MYH10 expression at Day 30 and a decline in MYH9 at Year 1. These findings highlight the temporal dynamics of DiGs during ischaemic injury and recovery (Figure S7).

### 3.5 ceRNA Network Establishment

**FIGURE 6:**
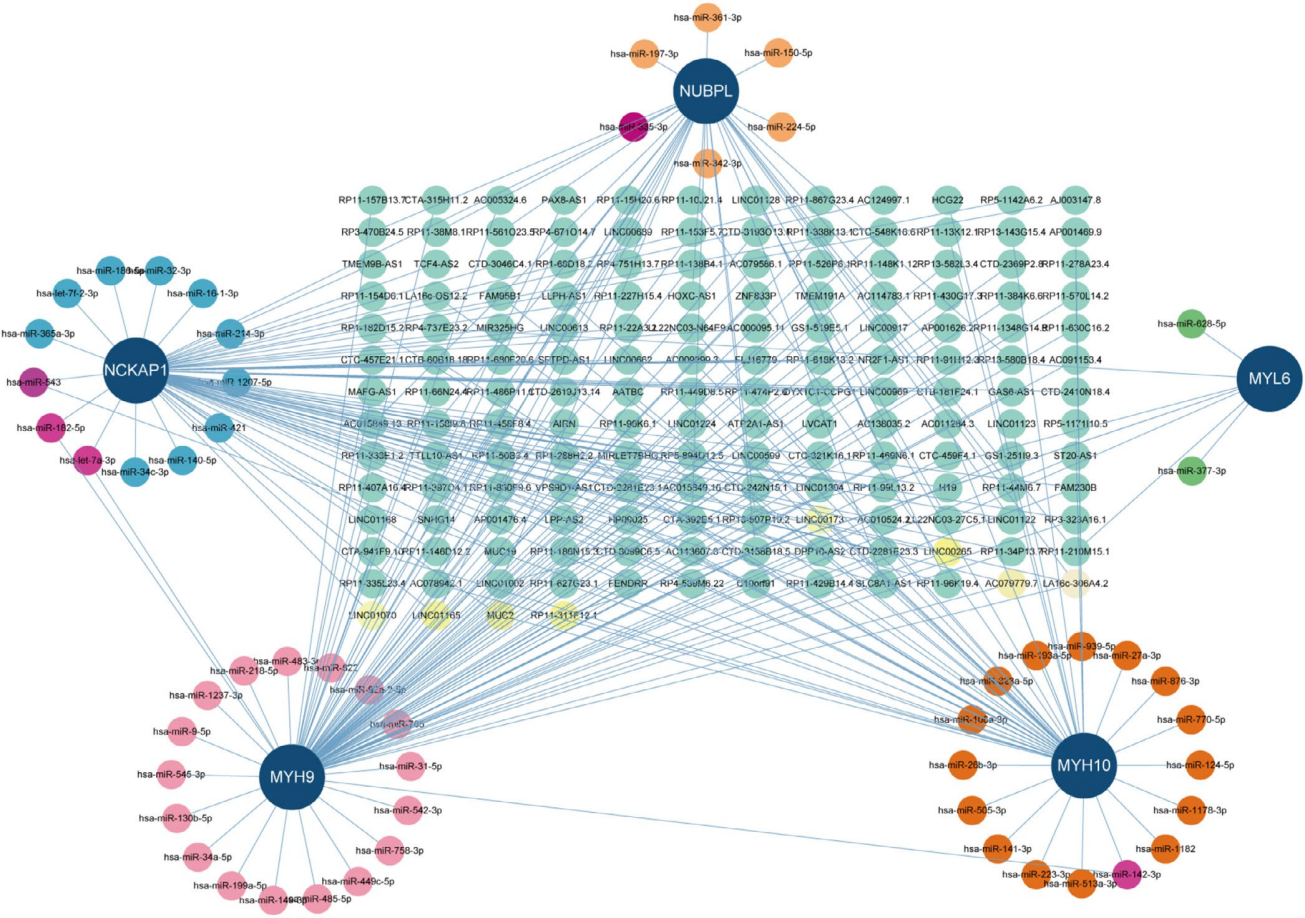
lncRNA–miRNA–mRNA regulatory network.

This network comprises 219 nodes, including 5 core diagnostic markers, 54 microRNAs (miRNA) and 160‐long noncoding RNAs (lncRNA), connected by 268 lines (Figure 6). In the ceRNA network, 73 lncRNAs can competitively bind to *Myh9* mRNA regulated by miRNAs such as hsa‐miR‐149‐3p, hsa‐miR‐142‐3p and hsa‐miR‐31‐5p, among which lncRNA LINC00265 can simultaneously target hsa‐miR‐485‐5p, hsa‐miR‐149‐3p and hsa‐miR‐449c‐5p. Additionally, 36 lncRNAs were observed to target *Myh10* mRNA, regulated by various miRNAs such as hsa‐miR‐141‐3p, hsa‐miR‐513a‐3p and hsa‐miR‐1178‐3p. Moreover, six lncRNAs were found to target *Myl6* mRNA, regulated by hsa‐miR‐377‐3p and hsa‐miR‐628‐5p. Additionally, 64 lncRNAs were identified to target *Nckap1* mRNA, regulated by various miRNAs, forming a complex network. Furthermore, 28 lncRNAs were observed to target *Nubpl* mRNA, regulated by various miRNAs such as hsa‐miR‐342‐3p, hsa‐miR‐224‐5p and hsa‐miR‐150‐5p. Notably, lncRNA l00969 was found to target miR‐1238‐3p, miR‐1207‐5p and miR‐484 simultaneously. The ceRNA network indicated the regulatory role of LINC01165, influencing the expression of NCKAP1, NUBPL and MYH9 (Figure 6).

### 3.6 Validation in a Single‐Cell Dataset

**FIGURE 7:**
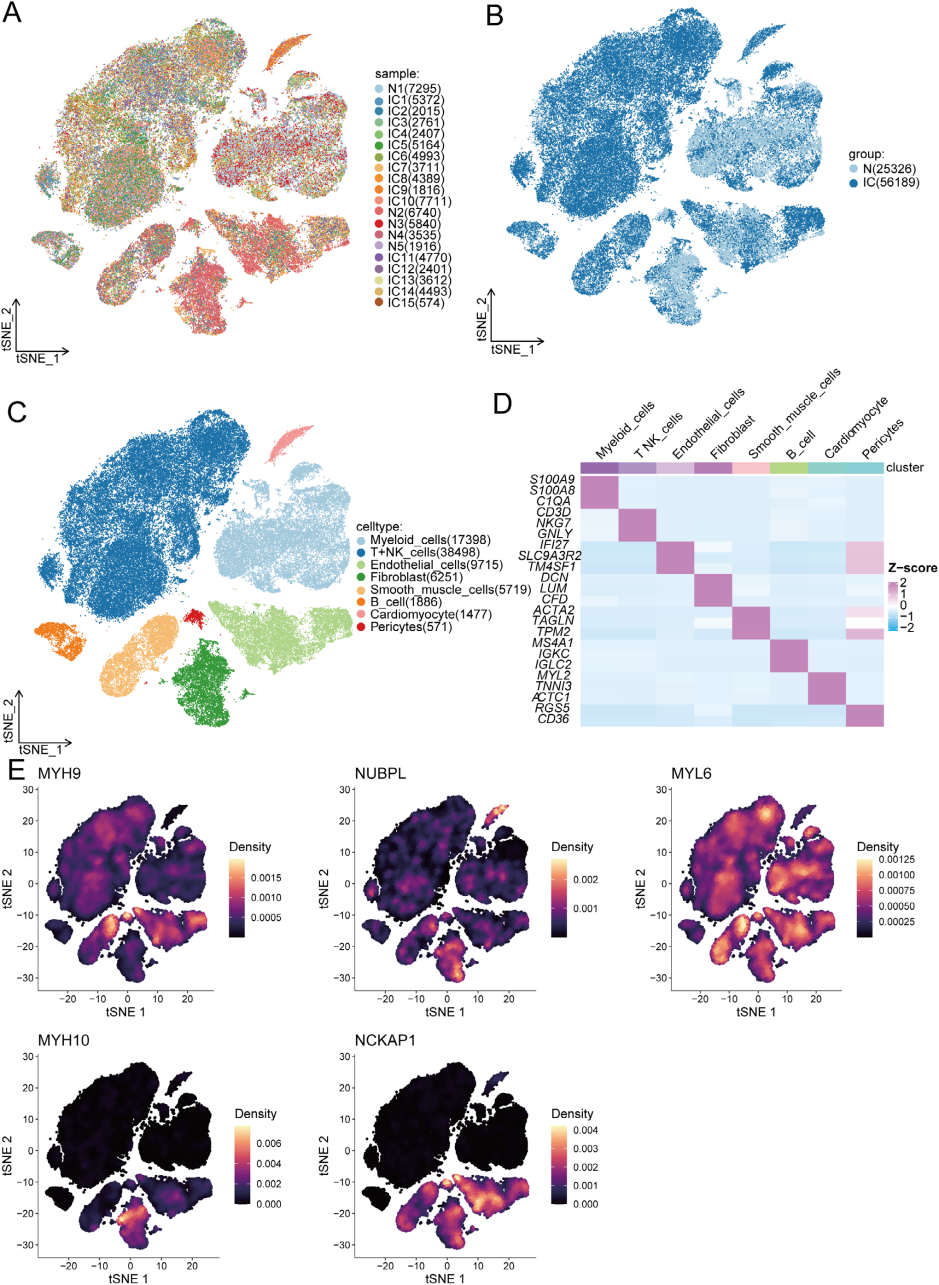
Single‐cell gene expression analysis in individuals with IC and normal controls. (A) t‐SNE dimensionality reduction plot of single cells from normal and IC samples. (B) t‐SNE dimensionality reduction plot of single cells from the normal and IC groups. (C) t‐SNE dimensionality reduction plot of eight cell types. (D) Heatmap displaying the marker genes for each cell type. (E) Expression density plots of five core genes (MYH9, NUBPL, MYL6, MYH10 and NCKAP1) across different cell types.

A total of 25,326 cells from healthy samples and 56,189 cells from IC samples passed established quality control standards across 20 samples (Figure 7A,B). Eight main cell types were identified within IC, including myeloid cells, T + NK cells, B cells, endothelial cells, fibroblasts, smooth muscle cells, cardiomyocytes and stromal cells (Figure 7C,D). Additionally, the expression distribution of five core DiGs (MYH9, NUBPL, MYL6, MYH10 and NCKAP1) across the eight cell types was analysed (Figure 7E). MYH9 exhibited high expression in smooth muscle cells, stromal cells and fibroblasts. NUBPL showed elevated expression in cardiomyocytes and fibroblasts. MYL6 displayed high expression in T + NK cells and myeloid cells. MYH10 was highly expressed in fibroblasts. NCKAP1 showed high expression in endothelial cells, fibroblasts and smooth muscle cells.

### 3.7 Five DiGs as Diagnostic Markers Were Validated in IC Animal Model

**FIGURE 8:**
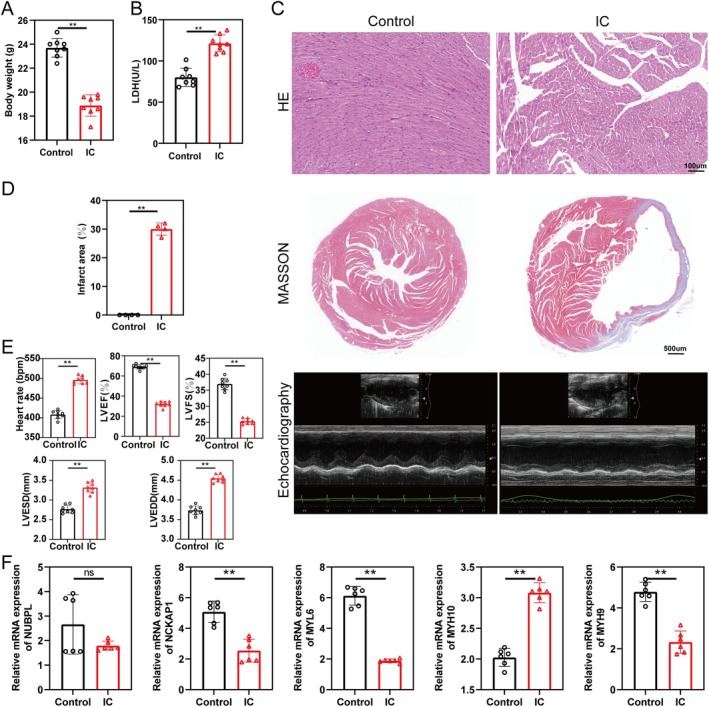
Male mouse data for myocardial injury in the IC model. (A) Changes in body weight between the Control and IC groups of mice (*n* = 8). (B) Changes in serum LDH levels between the control and IC groups (*n* = 8). (C) Representative haematoxylin and eosin (HE) staining of myocardial tissue showing histological changes in the control and IC groups. Scale bar = 100 μm. (D) Masson's trichrome staining depicting the myocardial infarction area. Scale bar = 500 μm. (E) Echocardiography results showing heart rate, left ventricular ejection fraction (LVEF), left ventricular fractional shortening (LVFS), left ventricular end‐diastolic diameter (LVEDD) and left ventricular end‐systolic diameter (LVESD) (*n* = 8). (E) Relative mRNA expression levels of NUBPL, NCKAP1, MYL6, MYH9 and MYH10 in male mice myocardial tissue (*n* = 6). Data are presented as mean ± SEM. (*p < 0.05, **p < 0.01.)

Compared to the control group, the body weight of male IC group mice significantly decreased 1 week postsurgery (Figure 8A) and serum LDH levels were significantly elevated in male IC group mice (Figure 8B). HE staining revealed disorganised myocardial cell arrangement, hypertrophy and abnormal fibre structures in both male and female IC group mice (Figures 8C and S8A). Masson staining showed a significant increase in myocardial infarction scar area in both male and female IC group mice (Figures 8D and S8B). Echocardiography results showed that LVEF and LVFS were significantly lower in both male and female IC group mice compared to the control group. Moreover, the IC group showed a marked increase in heart rate, LVEDD and LVESD (Figures 8E and S8C).

In female mice, mRNA expression of MYH10, MYH9, MYL6 and NCKAP1 was significantly downregulated in the IC group, while NUBPL expression was upregulated (Figure S8D). In male mice, compared to the control group, mRNA levels of MYH9, MYL6 and NCKAP1 were significantly downregulated, MYH10 was upregulated and NUBPL showed no difference (Figure 8F).

**FIGURE 9:**
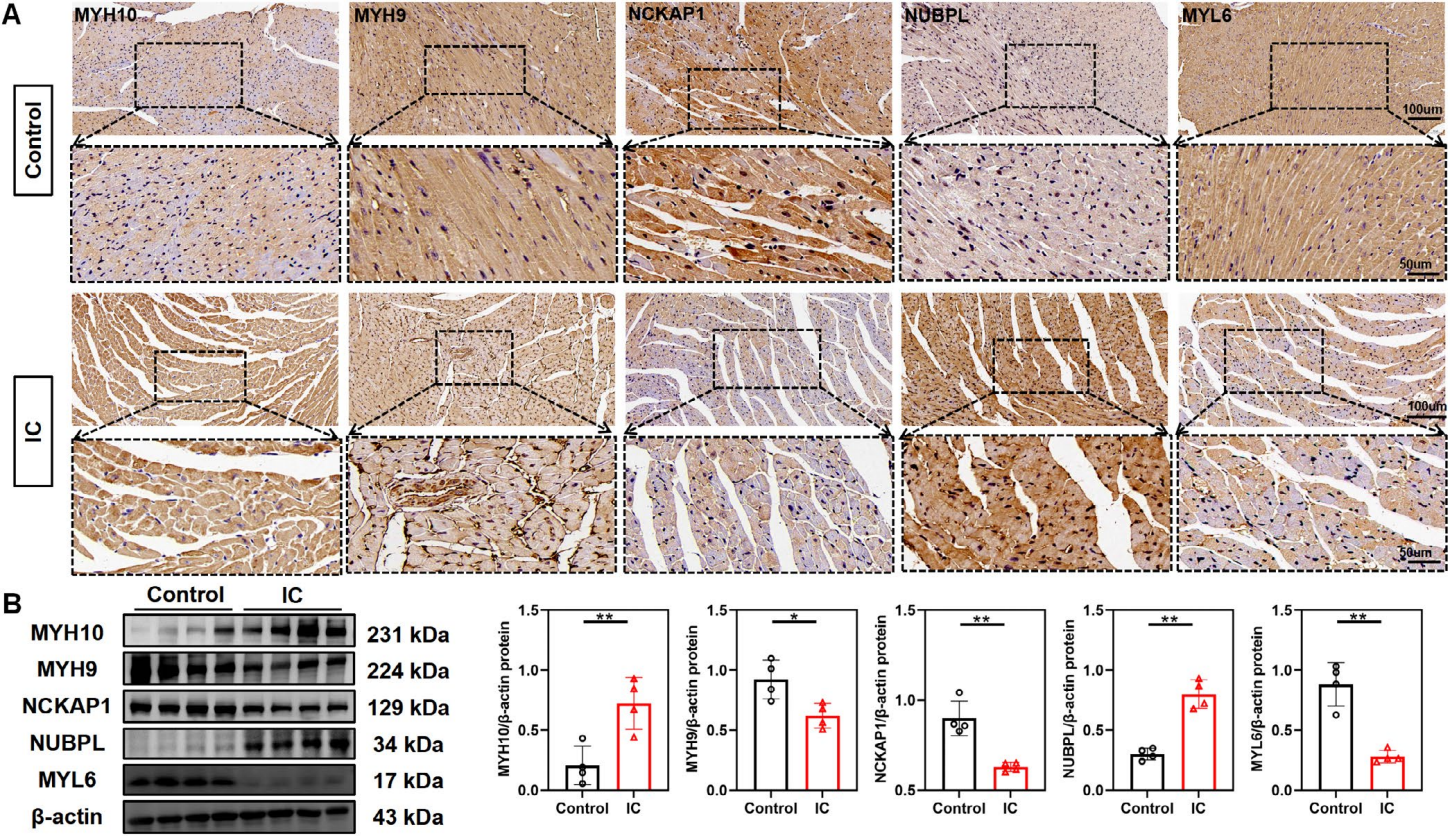
(A) IHC expression of MYH9, NUBPL, MYL6, MYH10 and NCKAP1 in myocardial cells of male mice in the control and IC groups (20× and 40×). (B) Western blot bands showing protein expression of MYH9, NUBPL, MYL6, MYH10 and NCKAP1 in male mouse myocardial tissue. (mean ± SD, *n* = 4). (*p ≤ 0.05, **p ≤ 0.01.)

IHC results indicated that, compared to the control group, the expression of NUBPL and MYH10 was significantly increased in the male IC group, while the expressions of MYH9, MYL6 and NCKAP1 were significantly decreased in the male IC group (Figure 9A). Furthermore, WB results were consistent with the IHC findings in the male IC group (Figure 9B). Compared to the control group, the expression of glucose metabolism‐related genes changed significantly in the male IC group, with GYS1 upregulated and HK2 and PYGM downregulated (Figure S9). HIF‐1α mRNA levels were significantly elevated in the male IC group (Figure S9).

## 4 Discussion

IC is a particular form of coronary heart disease resulting from prolonged myocardial ischaemia caused by coronary artery atherosclerosis. This condition leads to myocardial fibrosis, cardiac dysfunction and potentially heart failure [42]. In recent studies, the concept of disulfidptosis, a newly identified model of cell death, has emerged as a focal point of scholarly interest. The aetiology of IC remains incompletely understood, and disulfidptosis may have a potential involvement in its development. In this study, we aimed to elucidate the pathogenesis of IC by identifying a distinct cluster and developing a diagnostic model based on DiGs. Using bioinformatics analysis, we have identified MYH9, NUBPL, MYL6, MYH10 and NCKAP1 as potential key factors in the development of IC.

In animal studies, we observed a significant increase in the expression levels of NUBPL and MYH10 in myocardial tissue, while MYH9, MYL6 and NCKAP1 expressions notably decreased. IHC analysis of myocardial samples confirmed these findings, consistent with our preliminary bioinformatics analysis. Additionally, our results revealed significant alterations in glycogen metabolism‐related genes in IC. Specifically, GYS1 expression was upregulated in IC, while PYG and HK2 were reduced, suggesting enhanced glycogen synthesis, suppressed breakdown and impaired glycolysis. These metabolic shifts align with myocardial hibernation features [18, 19]. The upregulation of GYS1 may drive glycogen accumulation, a hallmark of myocardial hibernation, while the downregulation of PYG and HK2 further supports a shift towards glycogen storage over utilisation [18, 19]. This adaptation likely minimises energy consumption, protecting cardiomyocytes under ischaemic stress. Additionally, the observed glycogen accumulation and redox imbalance may link glycogen metabolism to disulfidptosis, a novel cell death pathway regulated by SLC7A11, which influences redox balance and cytoskeletal integrity [17]. These findings suggest that cardiomyocytes may adapt to changes in energy metabolism by regulating glycogen metabolism, thereby protecting myocardial tissue from further damage.

In the development of hypertension and atherosclerosis, inhibiting the formation of F‐actin and promoting the degradation of MYH9 protein can reverse vascular remodelling [43, 44]. The NUBPL gene belongs to the Mrp/NBP35 ATP‐binding protein family and is involved in nucleotide and ATP binding. NUBPL, as a key player in iron–sulphur cluster assembly, contributes to mitochondrial oxidative phosphorylation. Dysfunction of NUBPL may exacerbate energy deficits and oxidative stress, consistent with its role in heart failure and ischaemic cardiomyopathy progression [45, 46]. Previous studies have shown that reduced expression of MYL6 and MYH10 leads to decreased cardiac myofilament content and significantly reduced sarcomere length, indicating an essential role for MYL6 and MYH10 in cardiac development [47, 48]. Research has indicated that Tianxiandan significantly improves myocardial ischaemia by reducing the expression of MYH10‐related proteins [49]. Aberrant glycogen metabolism (alterations in MYL6 and NCKAP1 expression) may induce myocardial hibernation and activate the disulfidptosis pathway, leading to cell death and worsening of myocardial function [18, 19]. NCKAP1 has been identified as a new target gene regulated by miR‐214 in vascular smooth muscle cells. It plays a crucial role in mediating the effects of miR‐214 on the proliferation and migration of these cells [50]. These findings highlight the need to explore the interplay between DiGs and glycogen metabolism in IC to uncover novel therapeutic targets.

Similar to the role of disulfidptosis‐related genes in acute myocardial infarction mentioned in Huang et al. [51], our study also reveals the potential involvement of these genes in IC. However, in contrast to Huang et al. who focused on disulfidptosis in the context of acute myocardial infarction and its immune microenvironment, our research shifts the focus to the chronic setting of IC. Notably, we identified a novel association between glycogen metabolism dysregulation and disulfidptosis in IC—evidenced by the upregulation of GYS1 along with the downregulation of other key glycogen metabolism genes. This finding suggests that the adaptive myocardial hibernation observed in IC may be mediated, at least in part, by alterations in glycogen metabolism that interface with disulfidptosis pathways. Such a distinction not only broadens the understanding of disulfidptosis beyond acute myocardial injury but also underlines the diagnostic potential of our disulfidptosis‐related gene signature (MYH9, NUBPL, MYL6, MYH10 and NCKAP1) for early detection and therapeutic intervention in IC.

In light of the potential relevance of sex‐based differences in the pathophysiology of disulfidptosis, we repeated the IC model experiments in female mice and analysed the expression of key targets (MYH9, NUBPL, MYL6, MYH10 and NCKAP1). The results showed that gene expression patterns in female mice mirrored those in male mice, suggesting that the regulation of these genes in the context of ischaemic cardiomyopathy is consistent across sexes. This finding aligns with previous studies indicating that core pathways of myocardial injury may exhibit similar trends irrespective of sex. Based on the GSE57338 dataset, an analysis of the correlation between DiGs and gender and age revealed that the expression levels of MYH9 and MYL6 were significantly negatively correlated with age, which may indicate that age‐related myocardial metabolic or structural remodelling changes are associated with the regulation of these genes. However, the expression levels of other genes were not significantly associated with age or gender, indicating that their functions may not be influenced by clinical demographic variables. Further research with increased sample sizes is needed to investigate the specific mechanisms of how age and gender regulate gene expression.

During the ischaemic process of IC, the myocardium shows areas of coagulative necrosis and swelling, accompanied by the infiltration of inflammatory cells. Functional analysis revealed their association with immune responses and the progression of disulfidptosis, notably involving naive B cells, resting NK cells, activated NK cells and M2 Macrophages. Utilising these 17 disulfidptosis genes, we classified all samples into clusters, namely, Cluster 1 and Cluster 2. Cluster‐1 exhibited higher immune cell infiltration compared to Cluster 2, suggesting a stronger immune characteristic in Cluster 1. The core genes are mainly distributed in cardiomyocytes, fibroblasts, B cells, smooth muscle cells and endothelial cells, related to T + NK cells, B cells and macrophages, indicating that the expression of core genes in IC cardiomyocytes may be associated with immune cell infiltration.

Recent studies have demonstrated that the immune system is crucial in the onset and progression of IC. M2 macrophage polarisation can reduce myocardial fibrosis caused by IC and reverse ventricular remodelling [52, 53]. During the occurrence of IC, the activation of B cells and NK cells can inhibit the maturation and trafficking of inflammatory cells, thereby altering local cytokines and reducing damage to the myocardium [54, 55, 56]. Concurrently, our investigation revealed noteworthy distinctions in immune cells between the two identified clusters. Consequently, forthcoming research ought to prioritise the exploration of the intricate relationship and fundamental mechanisms between DiGs and immune cells in the context of IC.

MicroRNA (miRNA) has emerged as a pivotal epigenetic regulator in IC‐related genes, and advancements have been achieved in miRNA‐based biomarkers and their therapeutic implications [57]. Consequently, a ceRNA network employing five core diagnostic markers was constructed to explore the regulatory mechanisms governing these markers. The ceRNA network analysis unveiled the interaction of hsa‐mi‐543 with MYH10 and NCKAP1. Inhibiting the expression of hsa‐mi‐543 and hsa‐mi‐182‐5p can alleviate myocardial ischaemia–reperfusion injury [58, 59]. Relevant studies suggest that miR‐335‐3p can serve as a diagnostic biomarker for acute myocardial infarction and heart failure [60, 61, 62].

However, our study has several limitations. First, the sample size was limited, and validation with independent multicentre cohorts was lacking. Larger‐scale clinical data are required in the future to further verify the expression levels of DiGs. Second, this study primarily focused on the terminal stage of IC and failed to dynamically assess the temporal expression patterns of DiGs during disease progression. Future validation can be conducted using longitudinal animal models and clinical time‐series data. Furthermore, although this study proposed a prediction model based on DiGs, its clinical application requires integration with detailed prognostic data and cost‐effectiveness analysis to assess its feasibility and practicality. Most importantly, our study did not include a regional assessment of cardiac apoptosis, such as comparing apoptotic markers between infarcted and remote areas of the left ventricle. Given that previous studies have reported significant regional variations in apoptosis, this represents a notable limitation, as it restricts our understanding of the spatial heterogeneity of cell death mechanisms in IC. Future studies should incorporate techniques such as TUNEL staining and immunohistochemistry to comprehensively evaluate regional apoptotic differences, thereby providing deeper insights into the interplay between disulfidptosis and other forms of cell death. Finally, combining molecular biomarkers with traditional diagnostic tools (e.g., electrocardiography and imaging) may offer more cost‐effective strategies for early diagnosis and precise treatment of IC.

## 5 Conclusions

This study highlights the diagnostic potential of disulfidptosis‐related genes (DiGs) in IC. By integrating bioinformatics, machine learning and experimental validation, we identified five key genes—MYH9, NUBPL, MYL6, MYH10 and NCKAP1—as core diagnostic markers. These genes demonstrated distinct expression patterns in IC, influencing myocardial structure, immune cell infiltration and glycogen metabolism. The five‐gene–based SVM diagnostic model exhibited high predictive accuracy across multiple datasets, supported by experimental validation in IC mouse models. Furthermore, our findings suggest a strong link between glycogen metabolism dysregulation and disulfidptosis in IC. These results provide novel insights into the pathogenesis of IC and offer valuable molecular targets for early diagnosis, prognosis evaluation and therapeutic intervention.

## Author Contributions

Y.Z., J.T. and H.F. designed the study, S.X., G.Z., Y.Z., X.T., Z.Q., F.Y., J.F. and X.B. conducted the experiments and data analysis. S.X., Y.Z. and X.T. wrote and revised the manuscript. All authors read and approved the final manuscript.

## Ethics Statement

All animal experiments were performed with the approval of the Institutional Animal Care and Use Committee of The First Affiliated Hospital, Zhengzhou University, China (Protocol# 20231022013).

## Conflicts of Interest

The authors declare no conflicts of interest.

## Supporting information


Data S1.

## Data Availability

The datasets used and/or analyzed during the current study are available from the corresponding author on reasonable request.
