# Supplementary material for: A Novel Disulfidptosis‐Related Diagnostic Gene Signature and Differential Expression Validation in Ischaemic Cardiomyopathy
Source: J Cell Mol Med. 2025 Mar 11;29(5):e70475. doi: 10.1111/jcmm.70475 (PMC11897062; doi:10.1111/jcmm.70475)
Supplement: Supplementary file 1 — Data S1. [file JCMM-29-e70475-s001.docx]

**Supplementary Material**

**1. Supplementary Figures**

**
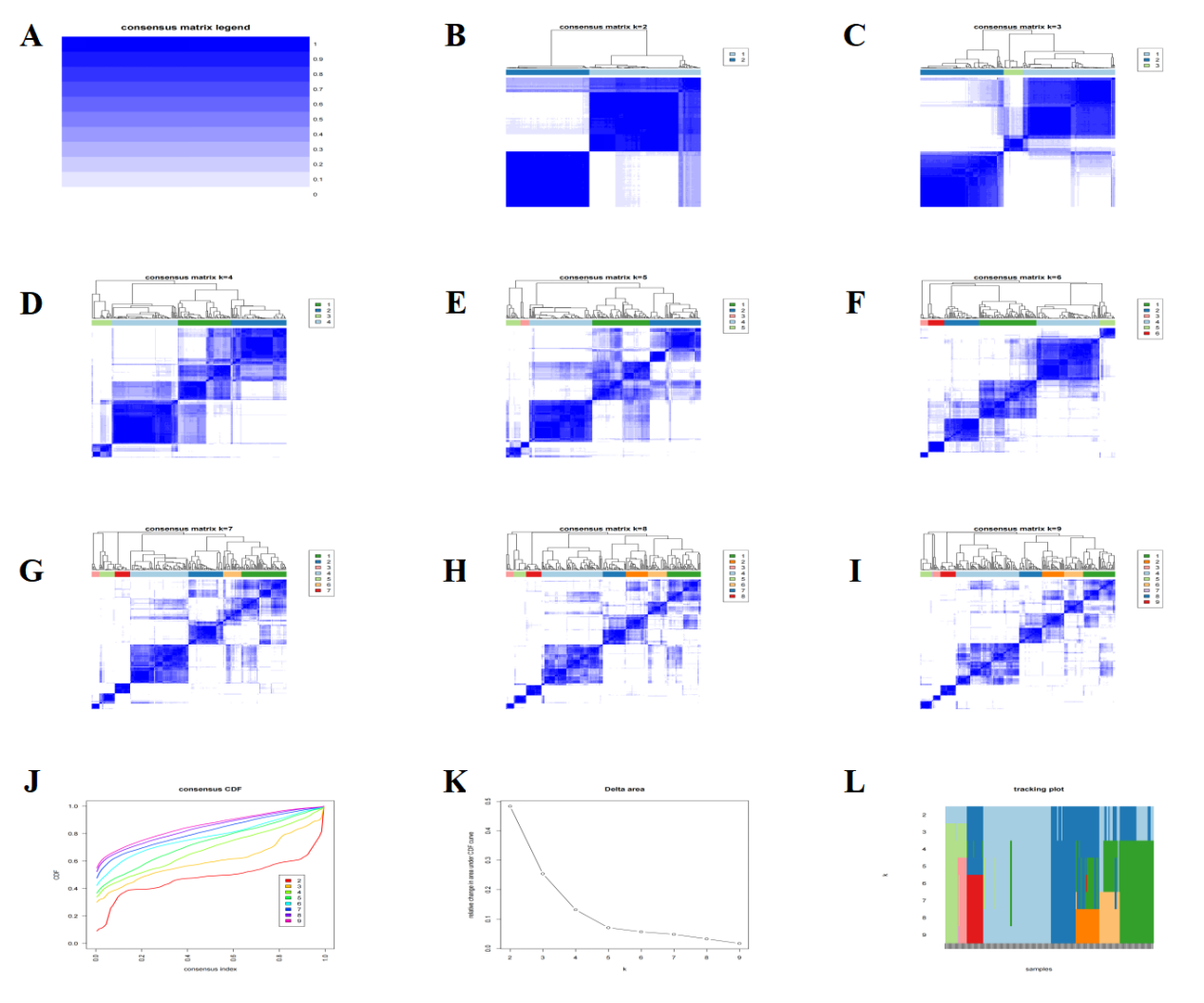
**

**Supplementary Figure 1**: Identification of disulfidptosis-related molecular clusters in IC. (A-I) Consensus clustering matrix when k = 2. (J) Uniform clustering cumulative distribution function (CDF) with k from 2 to 9. (K) The change of area under CDF curve with k from 2 to 9. (L) The tracking plot showed the relationship between samples and clusters.


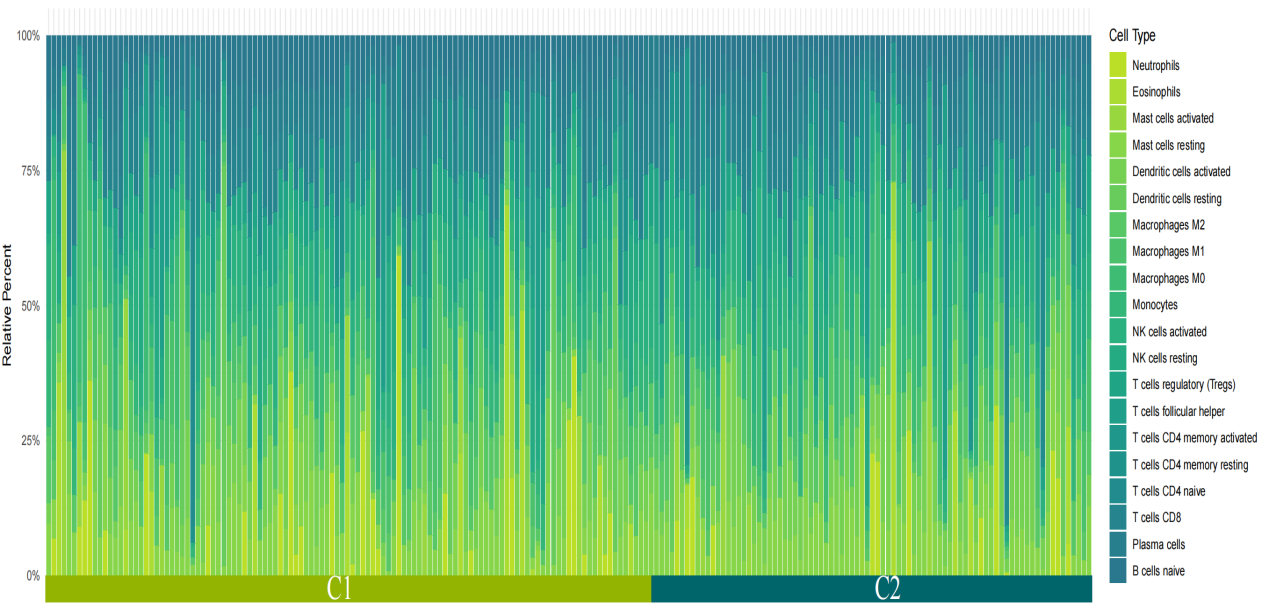


**Supplementary Figure 2:** The relative abundances of 22 infiltrated immune cells

between two disulfidptosis clusters.


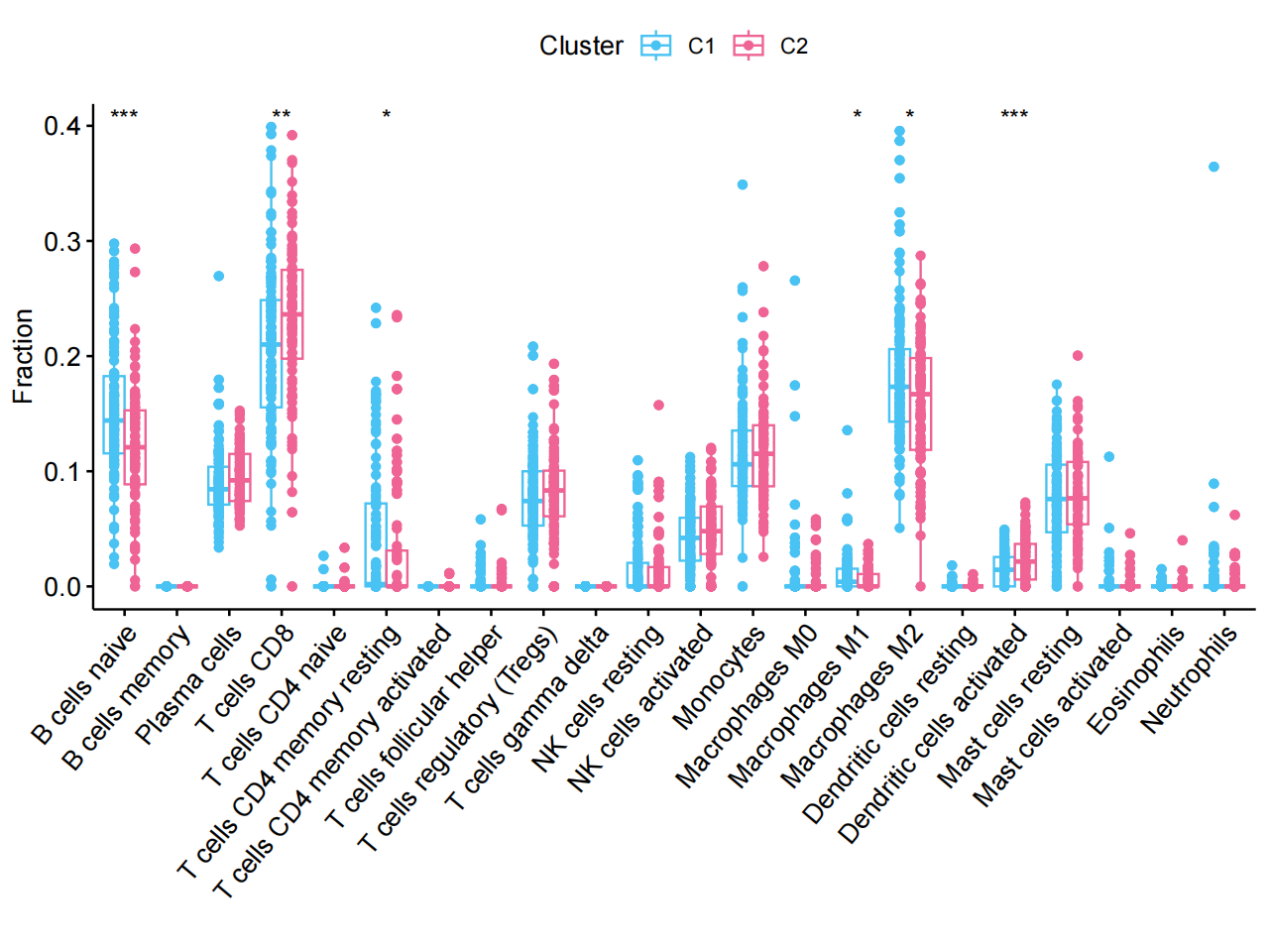


**Supplementary Figure 3:** Boxplots showed the differences in immune infiltrating

between two disulfidptosis clusters. *p < 0.05, **p < 0.01.


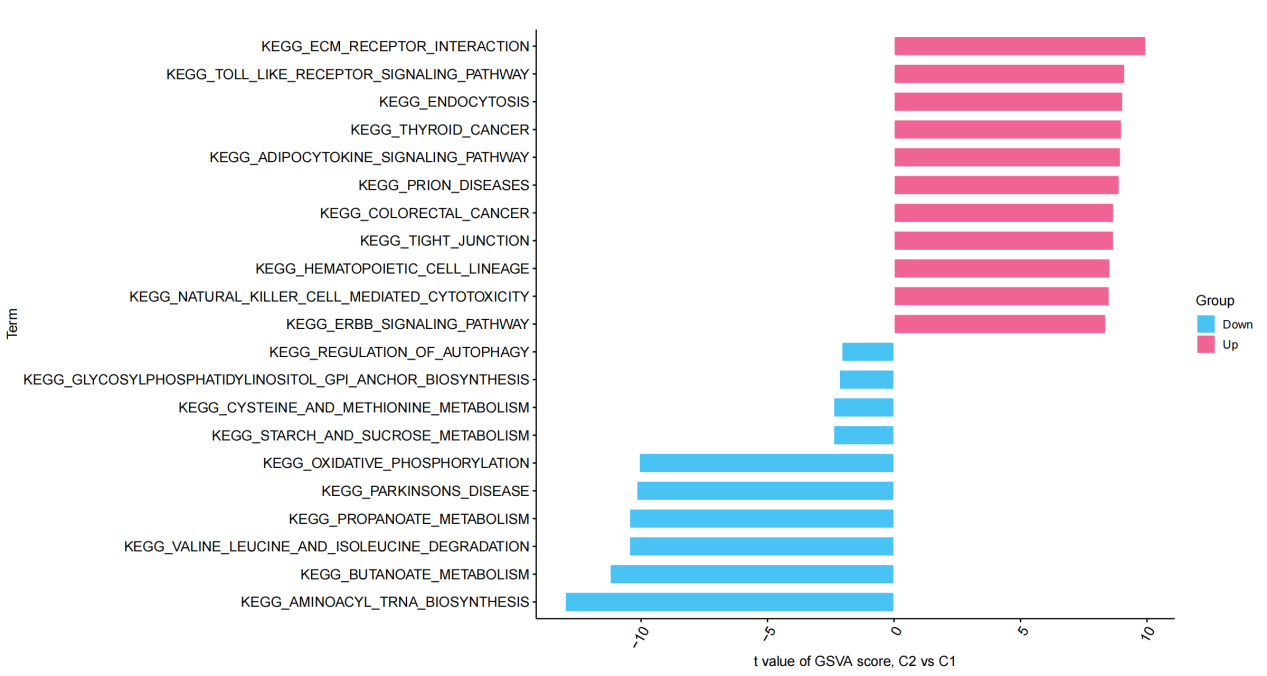


**Supplementary Figure 4:** Differences in hallmark pathway activities between cluter1

and cluster2 samples ranked by t-value of GSVA method.


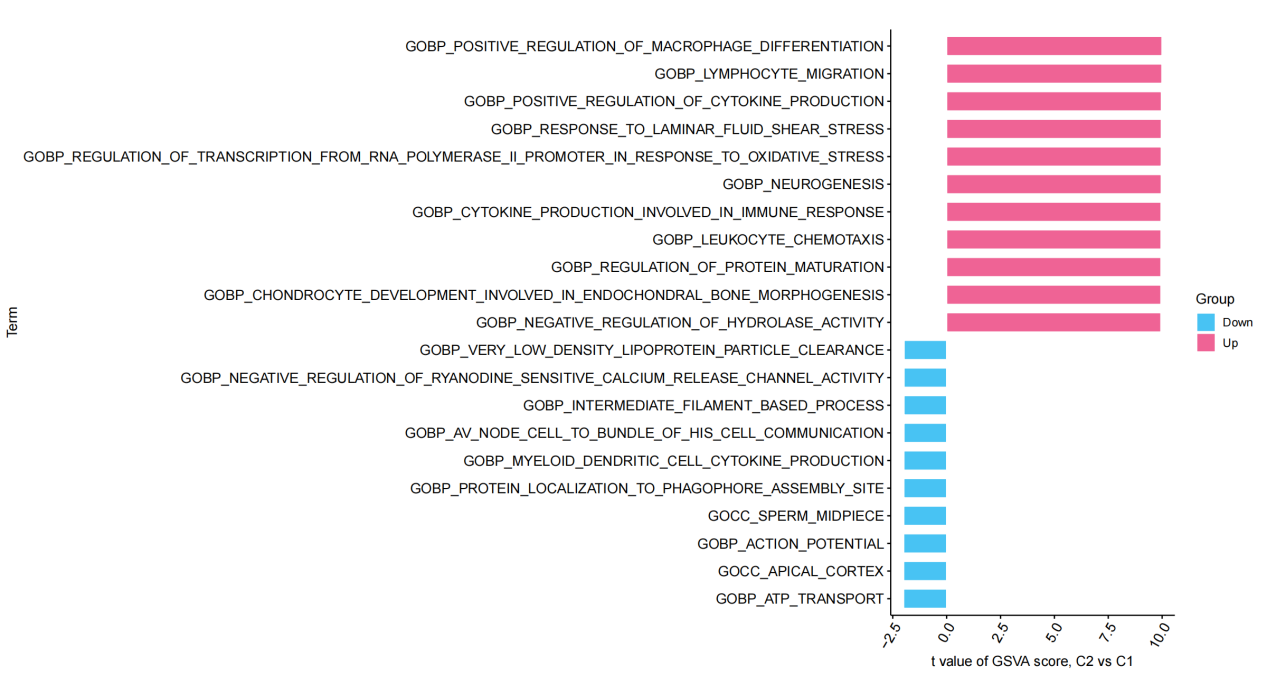


**Supplementary Figure 5:** Differences in biological functions between cluter1 and

cluster2 samples ranked by t-value of GSVA method.


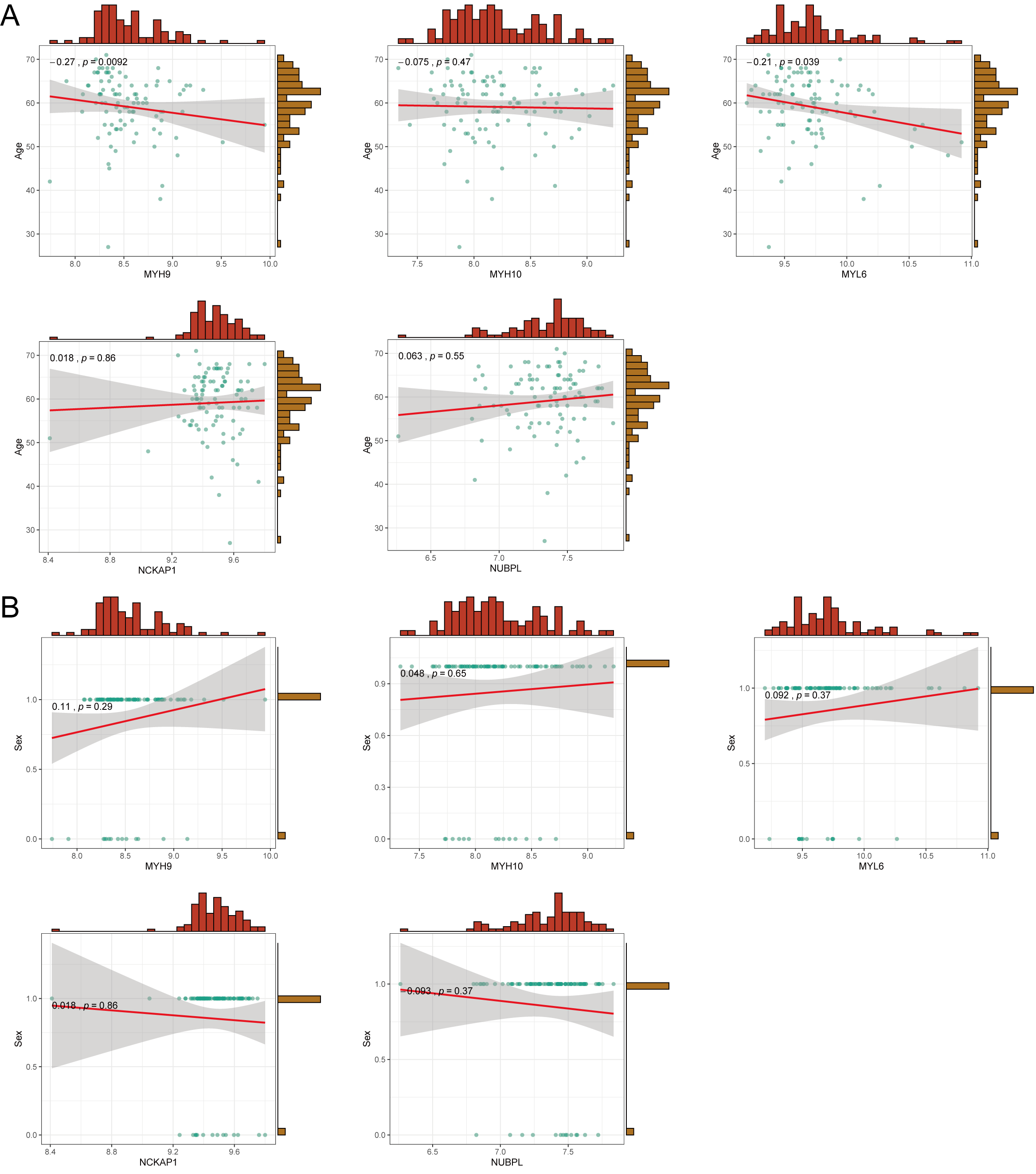


**Supplementary Figure 6:** (A) Linear correlation between the expression levels of MYH9, MYH10, MYL6, NUBPL, and NCKAP1 genes and sample age. (B) Correlation between the expression levels of MYH9, MYH10, MYL6, NUBPL, and NCKAP1 genes and sample gender.


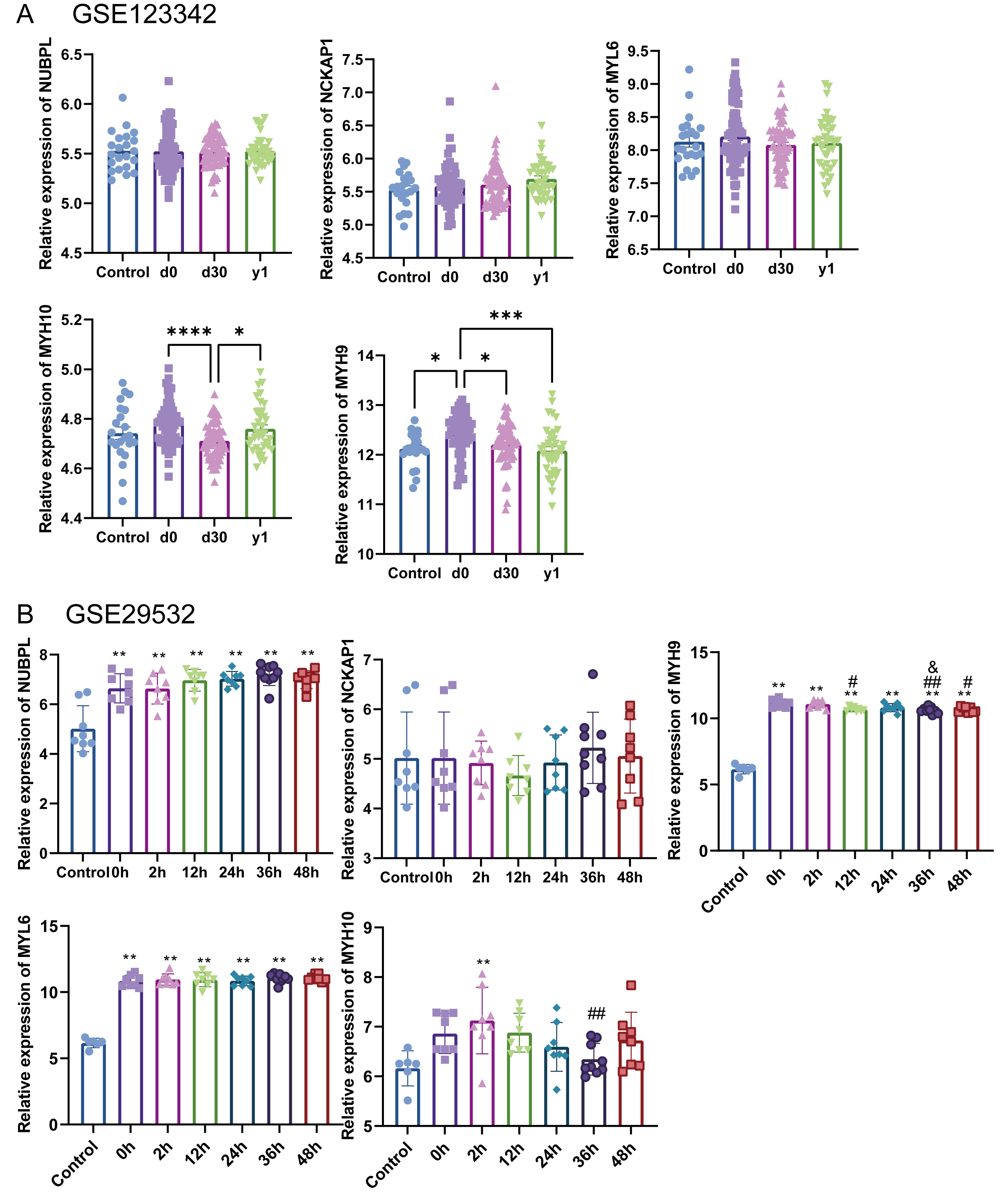


**Supplementary Figure 7:** (A) In the GSE123342 dataset, the expression levels of five genes (NUBPL, NCKAP1, MYL6, MYH10, and MYH9) were analyzed at control (Control), day 0 (d0), day 30 (d30), and year 1 (y1) post-myocardial infarction (MI). Statistical significance was assessed using one-way ANOVA (*p < 0.05, **p < 0.01, ***p < 0.001, ****p < 0.001). (B) In the GSE29532 dataset, temporal expression profiles of the five genes were analyzed at multiple time points (0h, 2h, 12h, 24h, 36h, and 48h) post-acute MI. Data are presented as mean ± SEM. Statistical significance: Compared to the Control group, **p < 0.01. Compared to the 0h group, ^#^p < 0.05, ^##^p < 0.01. Compared to the 2h group, ^&^p < 0.05.


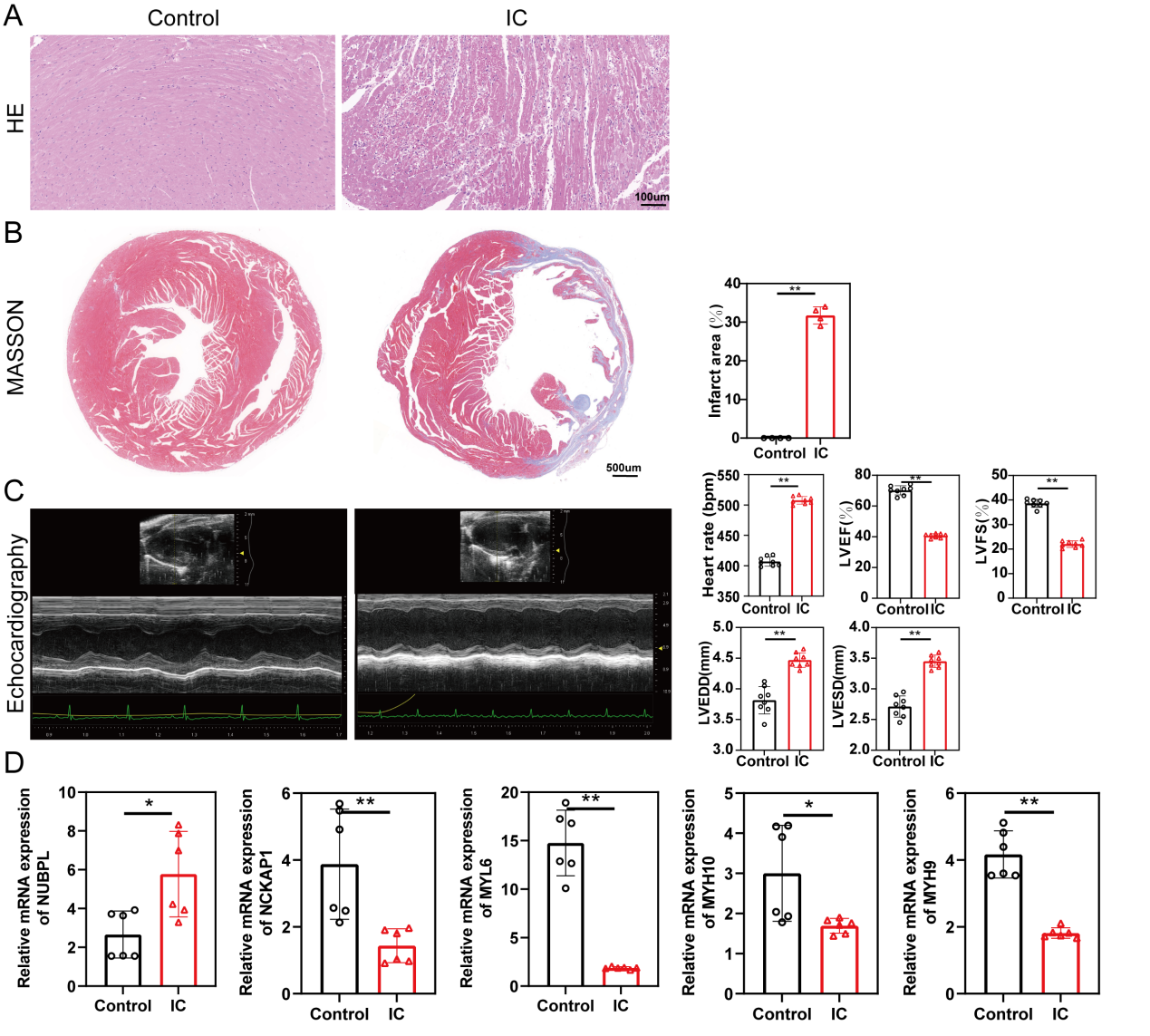


**Supplementary Figure 8:** Female Mouse Data for Myocardial Injury in the IC Model. (A) Representative hematoxylin and eosin (HE) staining of myocardial tissue showing histological changes in the control and IC groups. Scale bar = 100 μm. (B) Masson’s trichrome staining depicting the myocardial infarction area. Scale bar = 500 μm. (C) Echocardiography results showing heart rate, left ventricular ejection fraction (LVEF), left ventricular fractional shortening (LVFS), left ventricular end-diastolic diameter (LVEDD), and left ventricular end-systolic diameter (LVESD) (n=8). (D) Relative mRNA expression levels of NUBPL, NCKAP1, MYL6, MYH9, and MYH10 in female mice myocardial tissue (n=6). Data are presented as mean ± SEM. Statistical significance: *p < 0.05, **p < 0.01.


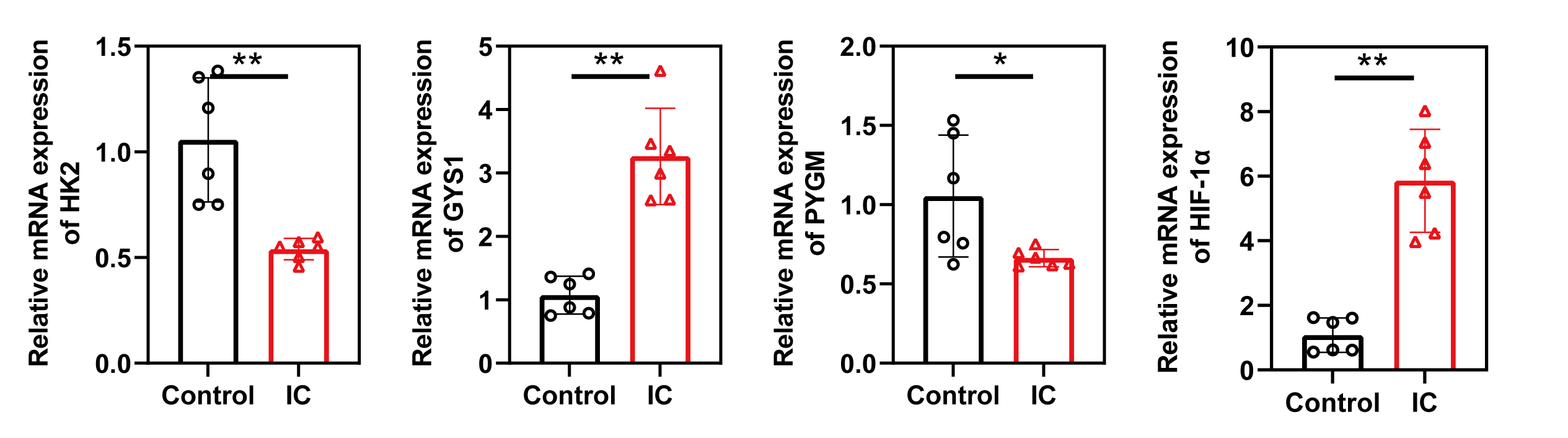


**Supplementary Figure 9:** mRNA expression levels of glucose metabolism-related genes (HK2, GYS1, PYGM) and the hypoxia marker gene (HIF-1α) in myocardial tissues of male IC and control groups (n=6). Data are presented as mean ± SEM (n=6). Statistical significance: *p < 0.05, **p < 0.01.

**
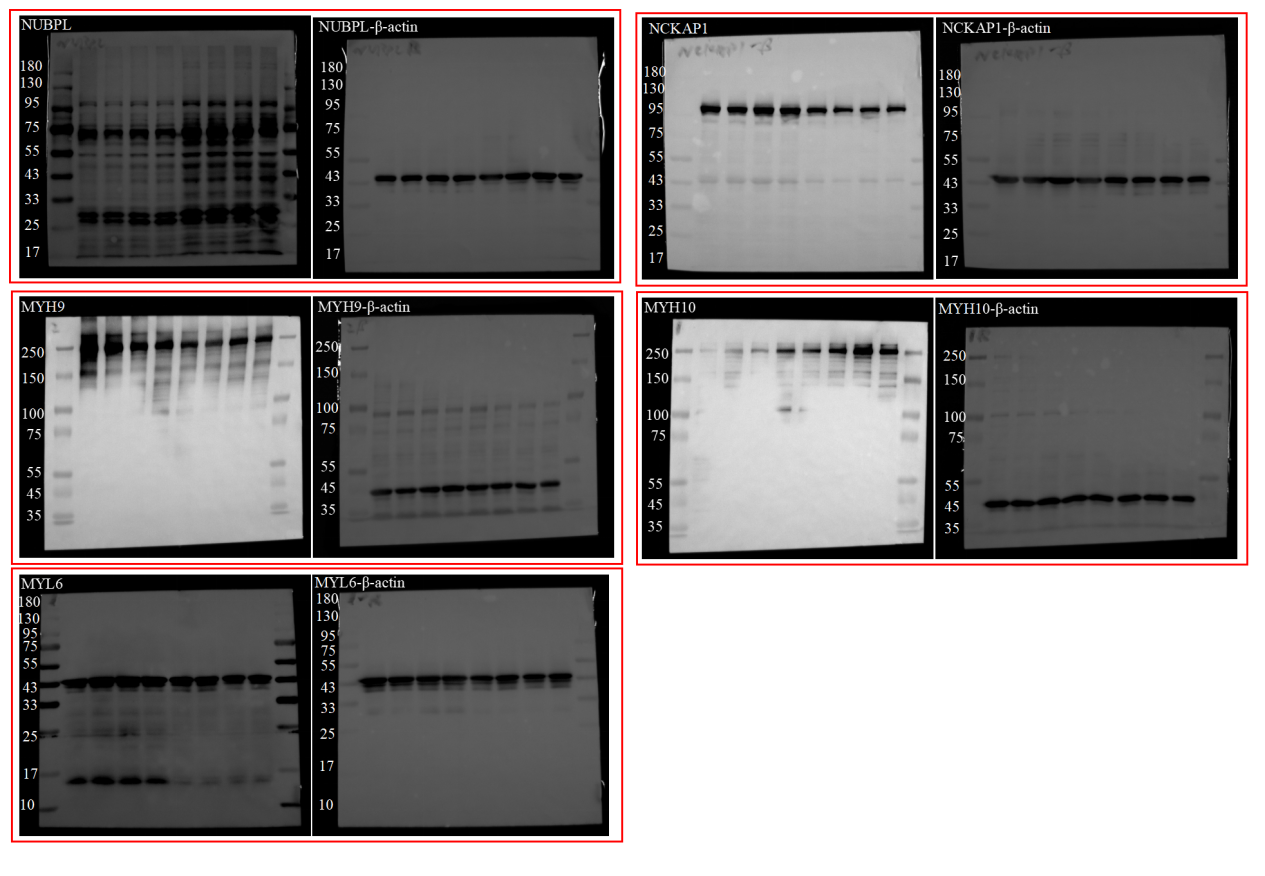
**

**Supplementary Figure 10:** uncropped Gels and Blots images.

**2. Supplementary Tables**

**Supplementary Table 1. Detailed GSE data for each group**

**Table S1. Detailed GSE data for each group**

| GEO ID | Platform | Samples | Number of Controls | Number of Cases | Country | Year | Author |
| --- | --- | --- | --- | --- | --- | --- | --- |
| Training set | | | | | | | |
| GSE5406 | GPL96 | 124 | 16 | 108 | USA | 2006 | Thomas Cappola |
| GSE57338 | GPL11532 | 231 | 136 | 95 | USA | 2014 | Michael Patrick Morley |
| Validation set | | | | | | | |
| GSE29532 | GPL5175 | 55 | 7 | 48 | Spain | 2011 | Francisco García García |
| GSE123342 | GPL17586 | 192 | 22 | 170 | Belgium | 2018 | Maarten Vanhaverbeke |
| GSE57345 | GPL9052 and GPL11532 | 195 | 134 | 61 | USA | 2014 | Michael Patrick Morley |
| GSE48166 | GPL9115 and GPL9442 | 31 | 16 | 15 | USA | 2013 | LIGUO WANG |
| GSE145154 | GPL20795 and GPL24676 | 20 | 5 | 15 | CHINA | 2021 | Shengshou Hu |
